# Strength from Within: Reversible Reinforcement of Paper through In-Sheet Formation of Thiol-Catechol Polymers

**DOI:** 10.1021/acsami.5c13781

**Published:** 2025-10-27

**Authors:** Lukas D. Bangert, Nicole Kirchner, Ching-Yi Choi, Markus Biesalski, Hans G. Börner

**Affiliations:** † Department of Chemistry, Laboratory for Organic Synthesis of Functional Systems, 9373 Humboldt-Universität zu Berlin, Brook-Taylor-Str. 2, 12489 Berlin, Germany; ‡ Ernst-Berl-Institut Macromolecular and Paper Chemistry, 26536 Technical University Darmstadt, Peter-Gruenberg-Str. 8, 64287 Darmstadt, Germany

**Keywords:** cellulose paper materials, mussel glue polymers, paper process, wet strengthening additives, paper recycling

## Abstract

Mechanical integrity of paper materials is crucial for many applications, yet reinforcement strategies with polymer strengthening agents that interact strongly often result in inhomogeneous coatings. In-sheet polymerization and adhesion (InSPA) methodology enables the in situ generation of reinforcing polymers, facilitating their uniform deposition on the cellulose fibers despite high interaction capabilities, and significantly increasing the dry and wet strength of commercial filter papers. A set of polymers containing highly adhesive thiol-catechol-connectivities (TCC) is prepared from *o*/*m*/*p*-isomers of benzenedithiol and bisquinone A by the InSPA thiol-quinone Michael-type polyaddition route. During polymerization, the interaction capabilities of the TCC-polymers are built up gradually, enabling effective cellulose fiber penetration and homogeneous fiber coating. Polymer loadings of up to 48 wt.-% are reached, transferring rigidity and hydrophobicity to the paper fibers without jeopardizing paper porosity. The noncovalently modified papers show a doubling in tensile index in the dry and a 7-fold increase in the wet state, paving the way for effective oil/water separation membranes, where end-of-life recyclability by removal of the polymer coating in a water-based process is demonstrated.

## Graphical abstract

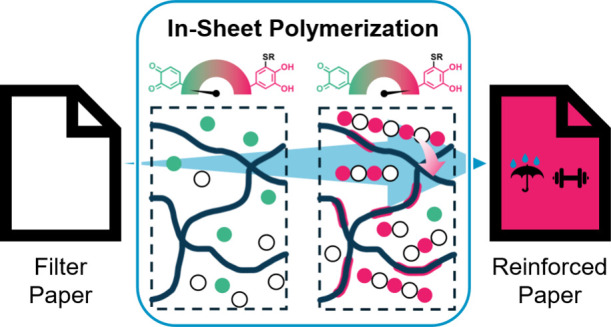

## 1 Introduction

The growing emphasis on environmental awareness in materials chemistry catalyzed a dramatic rise in interest toward sustainable reactions, processes, and materials.^1–3^ One of the most promising and versatile examples for a material platform made from renewable resources is paper. Depending on the intended use like packaging or printing, the properties of paper can be fine-tuned during the manufacturing process, making it a cost-effective, widely available and recyclable material.^4^

On the molecular level, paper is composed of a network of entangled semicrystalline cellulose fibrils, with fibers connected at joints through noncovalent interactions such as hydrogen bonding and London dispersion forces.^5^ Commercial formulations may include fillers and additives to enhance specific features like printability, while the abundance of accessible hydroxyl groups on cellulose enables facile chemical modification.^6–10^ These factors make paper very flexible - both chemically and mechanically - and have led to the development of highly advanced cellulose and paper materials with a myriad of uses reaching from unpowered analytical and diagnostic devices^11,12^ over 3D cell scaffolding^13^ to photonic pigments.^14^ For instance, Xi et al. realized a fully rewritable paper based on printing with pure water,^15^ while Walther and co-workers achieved cyclic adjustment of mechanical properties by electrothermal methods.^16^

However, despite its incredible versatility and the inherent robustness of cellulose fibers, the comparatively weaker fiber–fiber interactions in the network render paper prone to mechanical failure, particularly at low sheet thicknesses and under wet conditions. To address this shortcoming, both mechanical^17^ and chemical modifications of fibers have been intensively investigated, with the latter leading to the development of several classes of industrial additives such as polymeric wet strength and sizing agents.^18,19^ In addition, fiber premodification, e.g. by beating, can increase the bonded area and thereby improve paper dry strength, although it has little effect on the tensile strength in the wet state.^18^ Improvements in mechanical strength can thus be achieved through increased network density^20^ or cross-linking of cellulose fibers.^21^

While reinforcement of paper with low-^22^ or high- ^23,24^ molecular weight cross-linking-agents has been reported, homogeneous distribution of polymer coatings within the paper requires rigorous optimization and is critically dependent on polymer size, as high molecular weight polymers often tend to accumulate at fiber cross sections or on paper surfaces.^25–27^ In coating strategies that utilize preformed polymer additives, the use of polymers with strong interactions would be preferred for bridging fiber–fiber crossings and paper mesh stabilization. However, these polymers often tend to form agglomerates and result in undesired pore blocking.

An improved distribution of polymer additives can be achieved by performing polymerizations in situ within the paper: So far, this approach has been mainly utilized with conductive polymers, such as poly­(pyrrole) or poly­(aniline), that both cause mechanical weakening of the paper.^28–30^ Thus, wet strength agents are usually introduced at the pulp stage, where e.g. polyamide-epichlorohydrin- (PAE) or melamine-formaldehyde- (MF) polymer resins have been established in industrial papermaking to covalently cross-link fibers, increasing fiber cohesion under both dry and wet conditions.^21^

Although highly effective, such permanent modification of the cellulose fibers severely impacts recyclability, undermining the circularity of the material.^31^ Though repulping might still be possible in some cases,^32^ recent strategies have focused on designing reversible wet strength-systems. These rely on reversible covalent cross-links, such as disulfide bonds^33^ or Schiff bases,^26^ which can be cleaved on demand to recover the cellulose fibers. Christenson et al. introduced a sacrificial primer polymer layer that is adsorbed noncovalently onto the fiber surface before the PAE wet strength agent is introduced for covalent cross-linking.^34^

Noncovalent strategies for wet strength enhancement remain underexplored,^35^ despite their widespread use in other areas of paper production such assizing.^19^ In these cases, the coating relies on softer multipoint interactions with the fibers, using e.g. pendent sugars or carboxyl functionalities for anchoring on cellulose.

Catechol functionalities, inspired by the adhesive system of the marine mussel (*Mytilus edulis*), appear to be highly compatible with cellulose surfaces, facilitating the formation of robust hydrogen bonds and enabling hydrophobic interactions.^36,37^ This renders them potent, strong, and resilient anchor groups for polymers, which could be of considerable interest as effective noncovalent wet strength agents. Recently an intriguing access route to catechol derivatives has been described, enabling the synthesis of polymers with highly adhesive thiol-catechol-connectivities (TCCs) via a vinylogous Michael-type polyaddition of thiols to electrophilic *ortho*-quinones.^38^ A platform of TCC-polymer adhesives has been made accessible,^39^ which could be used on various materials under dry and even harsh seawater conditions. The approach has proven modular and scalable, allowing for the use of various AA/BB and AB-type monomers derived from commodity monomers,^39,40^ telechelic polymers,^41^ peptides^42–44^ and the renewable biomacromolecule lignin.^45,46^ The highly reactive and often shelf-stable quinones can be generated from phenol derivatives by chemical,^39,46,47^ electrochemical^48^ or enzymatic means.^38,44^ TCC-polymers exhibit a wide range of glass transition temperatures (*T*_g_) between −32 and +139 °C, which can be readily adjusted by the choice of monomer.^39^ It seems to be noteworthy, that the highly adhesive TCC-functionality is generated during the polymerization process, as the catechol functionalities are not present in the starting compounds (i.e., dithiols and bisquinones). This paves the way for the in-sheet production of adhesives and might circumvent the nonuniform deposition of strongly binding polymeric strengthening agents required for the homogeneous reinforcement of paper materials.

Here, we introduce the in-sheet polymerization and adhesion (InSPA) methodology for production of strengthening TCC-polymers as a versatile method for reinforcing premade paper sheets (Figure 1). Hydrophobic, high-*T*_g_ TCC-polymers were generated and employed as uniform cellulose fiber coating to transfer their hydrophobicity and mechanical strength to the cellulose network. The polymer deposition occurred strictly along the fibers with no obstruction of the pore system, allowing for significant increases in tensile index, especially in the wet state. The hydrophobized paper was used in oil/water-separation experiments and the noncovalent strengthening mechanism allowed for complete removal of the coating and recycling of the underlying cellulose fibers.

**Figure 1:**
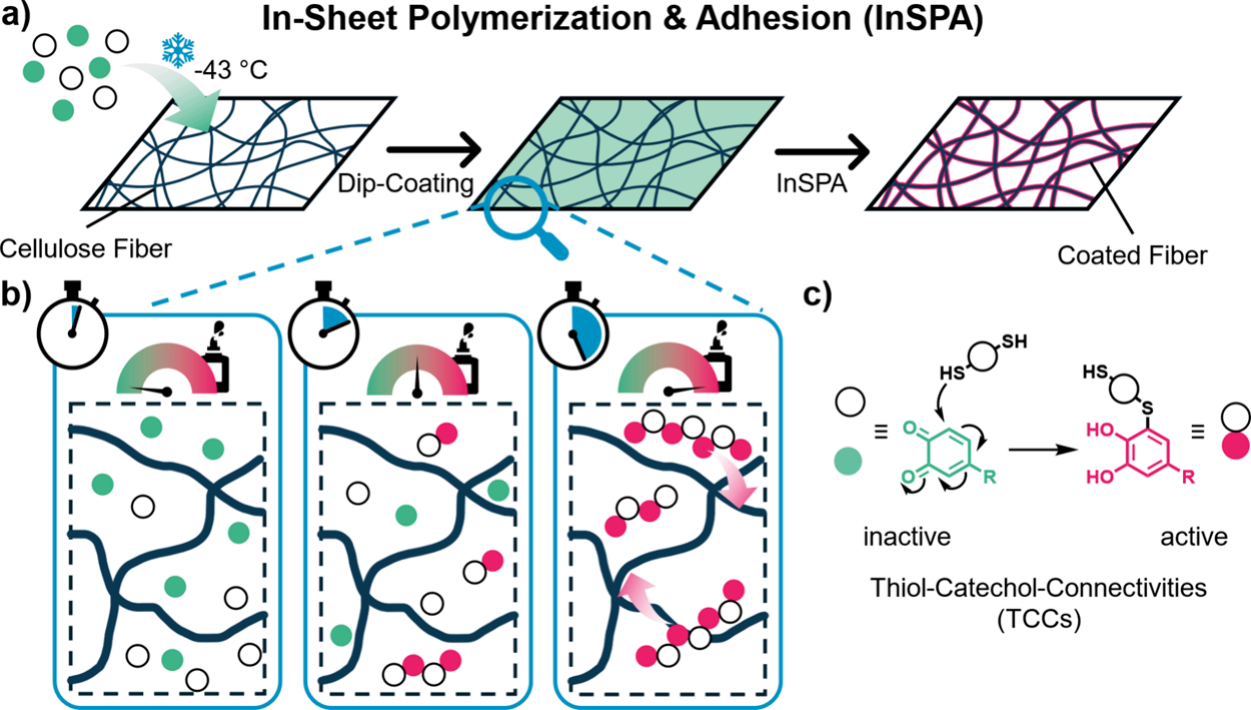
Concept of filter paper reinforcement via the InSPA methodology. Dip-coating of filter paper at low temperatures yields uniformly TCC-polymer-coated fibers (a). Adhesiveness of the coating increases gradually with molecular weight as the reaction progresses and leads to deposition of polymers onto the fibers (b). Mechanism of the Michael-like formation of thiol-catechol-connectivities (TCCs), transforming the inactive *ortho*-quinones (green) to adhesively active catechols (pink) (c).

## 2 Experimental Section

### 2.1 Materials

Oxone (monopersulfate compound), 2-iodobenzoic acid (98%), 2,6-ditertbutyl-4-methylphenol (BHT, >99%), 1,3-benzenedithiol (99%), and 2,2’-(ethylenedioxy)­diethanethiol (95%) were purchased from *Sigma-Aldrich Chemie GmbH* (Seelze, Germany). 1,4-benzenedithiol (>98%), bisphenol A (>99%), and Sudan IV were obtained from *TCI* (Tokyo, Japan). 1,4-Benzenedimethanethiol (95%) was obtained from *abcr* (Karlsruhe, Germany). Hydrochloric acid (37% in water) and sodium hydroxide (99%) were obtained from *Grüssing GmbH* (Filsum, Germany). 1,2-Benzenedithiol (97%), 1,4-benezenedithiol (97%), L­(+)-ascorbic acid (99%), and formic acid (FA, optima) were obtained from *Thermo Scientific* (Darmstadt, Germany). Deuterated dimethyl sulfoxide (DMSO-*d*_6_) was obtained from *deutero* (Kastellaun, Germany).*N*-Methyl-2-pyrrolidon (NMP) (peptide grade) was purchased from *Iris Biotech GmbH* (Marktredwitz, Germany). Tetrahydrofuran (≥99.8% for HPLC), *N*,*N*-dimethylformamide (DMF) (≥99.8%, for peptide synthesis), and acetonitrile (≥99.5%, for HPLC) were obtained from *VWR chemicals* (Dresden, Germany) and formic acid (98%) from *Acros Organics* (Geel, Belgium). MN 616 filter paper was obtained from *Macherey-Nagel* (Düren, Germany). Fluorescent Brightener 28 (Calcofluor White) was purchased from *MP Biomedicals* (Illkirch, France). Methylene blue was obtained from *Riedel-de Haën* (Seelze, Germany). Sodium ascorbate (≥99%) was purchased from *Fluka* (Buchs, Switzerland). ALBODUR 956 was purchased from *Alberdingk Boley* (Krefeld, Germany), DESMODUR N 3900 was purchased from *Covestro* (Leverkusen, Germany) and TIB KAT 318 was purchased from *TIB Chemicals AG* (Mannheim, Germany). All solvents not listed above were distilled prior to use. Iodoxybenzoic acid (IBX) was synthesized according to the procedure described by Schröter et al.^41^ Ultra pure water was produced using a SG LaboStar TM 1-UV system from *SG water* (Hamburg, Germany) with an Evoqua Water Technologies Polisher HP2 module ion exchanger (Electric conductibility 0.055 μS cm^–1^).

### 2.2 Instrumentation

#### 2.2.1 Confocal Laser Scanning Microscopy (CLSM)

All CLSM-measurements were carried out on a TCS SP8 with the corresponding software Leica Application Suite X (*Leica Microsystems*, Wetzlar, Germany). Free cellulose surfaces were stained with 100 μM aqueous Calcofluor White (CW) over 2 days. Excess dye was removed by rinsing with water and the paper strips were dried. The samples were subsequently embedded in epoxy resin (Desmodure 3900, Albodur 956, TIB Kat 318; 1:1:0.0005) and cut into 120 μm slices using a microtome. During the measurements, CW was excited at 405 nm and the fluorescence was detected between 420–470 nm. The fluorescent TCC-polymer was excited at 488 nm and fluorescence was detected between 520–660 nm. Around 50 Micrographs with 0.4 μm thickness were accumulated into maximum projections using ImageJ2 (1.54b).

#### 2.2.2 Confocal Raman-Microscopy

All Raman-spectra were obtained on an alpha300 R confocal Raman-microscope (*Witec*, Ulm, Germany) with 532 or 785 nm lasers and 10×, 20×, 50× and 100× lenses (*Zeiss*, Jena, Germany).

#### 2.2.3 Contact Angle Measurements

All contact angle measurements were carried out either on an OCA35 (*Dataphysics*, Filderstadt, Germany) or a Drop Shape Analyzer DSA25B (*Krüss*, Hamburg, Germany). Images were analyzed using the Young–Laplace-model in the corresponding software SCA20 and ADVANCE, respectively. Pure water or distilled cyclohexane were used in the experiments.

#### 2.2.4 Differential Scanning Calorimetry (DSC)

All measurements were performed on a DSC 3 system (*Mettler Toledo GmbH*, Gießen, Germany) connected to a TC100 cooler (*Peter Huber Kältemaschinenbau SE*, Offenburg, Germany). All measurements were performed under an argon atmosphere between 0 and 200 °C with a heating rate of 10 °C min^–1^. Samples of around 10 mg were measured in a punctured aluminum pan. *T*_g_ values were obtained from the second heating scan.

#### 2.2.5 Fourier Transform Infrared Spectroscopy (FT-IR)

All measurements were carried out on a Bruker Vertex 70v FT-IR spectrometer (*Bruker Optik GmbH*, Germany). Blank measurements were conducted before and after each sample.

#### 2.2.6 Mechanical Measurements

Qualitative bending tests were carried out on a Ta.XT.plus100C (*Stable Micro Systems*, Godalming, United Kingdom) using a 4,9 N force cell at 40–60% relative humidity after sample equilibration over at least 12 h. The samples were placed on two supports with an 18 mm gap in between and a perpendicular force was applied to each strip in the center of the gap. The initial (linear) force–distance-relationship was fitted by linear regression. T the resistance was averaged over the set and compared to the values for pristine MN 616 filter paper.

Quantitative tensile tests were carried out on a Zwick Z1.0 (*ZwickRoell GmbH* *& Co. KG*, Ulm, Germany) using a 100 N or 1 kN force cell according to ISO 1924-2 at 23 °C and 50% relative humidity. Sample sets consisted of five strips (1.5 × 12.0 cm). For analysis, textXpert II V3.71 was used, and the five individual values were averaged. For the determination of wet strength, the corresponding sample sets were soaked in water overnight, excess water was removed by briefly placing the paper strips on a paper towel, and the tensile strength was measured immediately thereafter. Dry and wet tensile indices were obtained by normalizing the tensile strength to the grammage. Relative wet strength was calculated as the ratio of wet to dry tensile index.

#### 2.2.7 Nuclear Magnetic Spectroscopy (NMR)

Liquid-state ^1^H and ^13^C NMR measurements were performed on a Bruker Avance II 500 or 600 MHz spectrometer (*Bruker BioSpin GmbH*, Rheinstetten, Germany) in the given deuterated solvent.

#### 2.2.8 Recycling Tests

Papers were dried to constant weight using a HC103 moisture analyzer (*Mettler Toledo*, Columbus, OH, USA). Sheet formation with the disintegrated fragments was performed on a Rapid-Köthen BB3 sheet former (*Haage Anagramm Technologien GmbH*, Peissenberg, Germany) in accordance with DIN EN ISO 5269-2.

#### 2.2.9 Size Exclusion Chromatography (SEC)

All measurements were carried out on an Eco-SEC-System with UV and RI-detection (HLC-8320 GPC) (*Tosoh*, Griesheim, Germany). Tetrahydrofuran (THF, HiPerSolv CHROMANORM for HPLC) was used as solvent and SDV columns (1000 Å 5 μm, 100,000 Å 5 μm and 1,000,000 Å 5 μm) from *PSS* (Mainz, Germany) were applied. The calibration was done using polystyrene calibration standards between 1480 g mol^–1^ and 3.04 × 10^6^ g mol^–1^ (*PSS*, Mainz, Germany).

#### 2.2.10 Thermal Gravimetric Analysis (TGA)

All measurements were carried out on a Thermogravimetric Analyzer Pyris 1 (*PerkinElmer*, Waltham, USA). Approximately 10 mg of each sample were weighed in a ceramic pan and heated from 30 °C up to 800 °C at a heating rate of 20 °C min^–1^ under argon atmosphere with a flow rate of 20 mL min^–1^.

#### 2.2.11 Ultraviolet/Visible (UV/vis) Spectroscopy

UV/vis-spectroscopy was performed on a Cary 60 spectrophotometer (*Agilent*, Santa Clara, USA) connected to a cryostat (*Unisoku Scientific Instruments*, Hirakata, Japan). Preserving the stoichiometric BQA/dithiol ratio of the InSPA process, kinetic measurements were taken at 20.0 ± 0.1 °C and −43.0 ± 0.1 °C using 10 × 10 mm quartz cuvettes by observing the *ortho*-quinone peak at 377.5 nm.

### 2.3 Methods

#### 2.3.1 Synthesis of Bisquinone A (BQA)

5.0 g of bisphenol A (20 mmol) were dissolved in methanol and mixed with 18.5 g of iodoxybenzoic acid (IBX) (66 mmol). The mixture was stirred for 15 to 30 min at room temperature and then cooled in an ice bath for 10 min. The solid was filtered off and washed with cold methanol. The colored solid was dissolved in chloroform and after filtration, the organic solvent was removed under reduced pressure. Analytical data was consistent with previous reports of our group.^39,41^ Yield: 4.1 g, 16.1 mmol, 81%.

#### 2.3.2 General Polymer Synthesis in Solution

BQA (1.5 mmol) was dissolved in DMF (10 mL), the solution was stirred, and 1.0 eq. of dithiol was added. The polymerization was carried out for 15 min at room temperature and the mixture was precipitated in a 3:2-MeOH:Milli-Q water mixture (120 mL). The precipitate was redissolved in acetone and precipitated in 120 mL of Milli-Q water. The precipitate was isolated and dried, and the TCC-polymers were obtained with a yield range of 75–90%.

#### 2.3.3 InSPA Paper Reinforcement Procedure

12.8 mg mL^–1^ of dithiol and 20.8 mg mL^–1^ BQA were separately dissolved in DMF. One set (5 strips, 5 × 1 cm) of MN 616 strips was prepared, cooled to −25 °C, and the two monomer solutions were cooled to −43 °C in an acetonitrile-nitrogen-slush. The monomer solutions were then mixed at −43 °C and the strips were immediately dipped into the mixture, agitated for approximately 1 s and subsequently removed. The wet strips were placed in a DMF-atmosphere to polymerize for 15 min and subsequently dried in air for 1 h at 90 °C. The dried strips could then be reused for further dipping cycles.

For the preparation of samples for tensile testing, the size of the paper strips was adjusted to meet the requirements of ISO 1924-2 (12 × 1.5 cm) and the soaking procedure was scaled up accordingly. For the preparation of the fluorescent sample P1*@FP ^**10**^, 1 mol-% of 1,4-BDT was replaced with the fluorescent dithiol DT*.
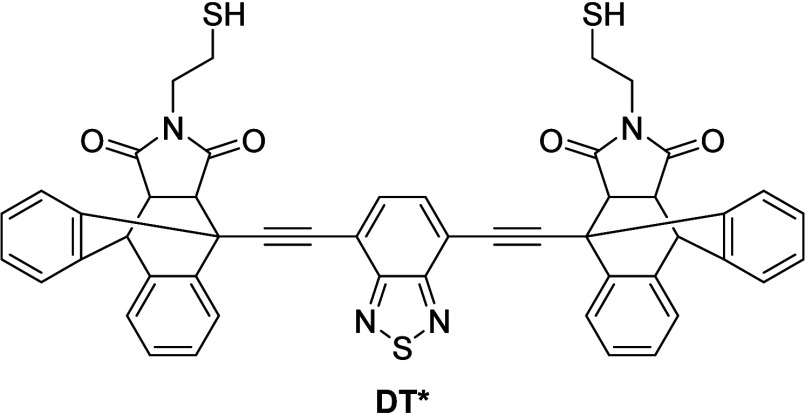


#### 2.3.4 Filtration Experiments

Initially, holes were cut into the screw caps of 15 mL centrifuge tubes to allow for the flow of liquid. Filter membranes were then placed between the screw caps and the threading, and the tubes were closed with the modified screw caps, sealing all other liquid pathways except for the membranes themselves. To enhance visibility, water was dyed blue with methylene blue and cyclohexane was dyed red with Sudan IV. The two liquids were then mixed vigorously.

In the vertical setup, a single tube was fitted with a TCC-membrane made of **P1@FP** ^**10**^ and placed with the membrane facing downward. A hole was cut into the tube at the topmost point and a stirring bar was added, followed by the mixture of cyclohexane and water. Upon vigorous stirring, only the percolation of cyclohexane through the membrane was observed, with the aqueous phase being retained above the filter.

In the horizontal setup, two centrifuge tubes were cut at the bottom and joined together by rubber tubing, forming a horizontal tube with two screw-capped exits. A hole was cut into the side of the tube, allowing for the addition of liquids. One exit was fitted with a TCC-membrane made of **P1@FP** ^**10**^ while the other was fitted with a membrane made of water-wetted and otherwise unmodified filter paper. Upon addition of the water/cyclohexane mixture, only cyclohexane percolation was observed at the TCC-membrane, while only water percolation was observed at the unmodified membrane.

#### 2.3.5 Recycling Experiments

The torn test strips from the mechanical tests were cut to dimensions 1.5 × 7 cm for subsequent recycling experiments or to dimensions 1.5 × 5 cm for gravimetrical analysis, respectively. The paper strips were divided into three groups and treated with deionized water, 0.22 M ascorbic acid in 1.25 M NaOH, or DMF, respectively, by immersion in 25 mL of solvent and shaking at 180 rpm for 2 h at room temperature. The solvent was then replaced with fresh solvent and the strips were shaken for an additional hour under the same conditions. After treatment, the paper strips were air-dried at room temperature.

The pretreated paper strips used in gravimetrical analysis were dried to constant weight at 105 °C and the weight was subsequently determined immediately after drying using an analytical balance. Due to rapid moisture uptake from ambient humidity, only the lowest recorded weight (directly after removal from the drying unit) was used for calculations, yet minor errors in sample mass cannot be ruled out.

The pretreated paper strips used in the repulping experiments were disintegrated in 200 mL of deionized water at room temperature using a mechanical stirrer at 900 rpm for 20 min. The resulting suspension was then diluted to 9 L and dewatered through a sheet former. Residual paper fragments retained on the forming sieve were then transferred to a carrier sheet and vacuum-dried for 12 min at 90 °C. The dried carrier sheets containing the dispersed fragments were photographed in transmission mode. The images were processed using Inkscape (version 1.3.2). Repulpability was evaluated based on the Dispersibility Score (DS) defined by Pfennich et al.,^49^ who provided a reference scoreboard. This scoreboard consists of 11 reference images corresponding to DS values from 0 (no visible disintegration) to 10 (complete disintegration). Each sample was evaluated in duplicate.

## 3 Results and Discussion

### 3.1 Solution and In-Sheet Polymerization of TCC-Adhesives

To evaluate suitable high-*T*_g_ polymers with adhesive TCC-functionalities for cellulose reinforcement, the previously described bisquinone A (BQA) was polymerized in solution with the conformationally constrained isomers 1,4-, 1,3-, and 1,2-benzenedithiol (BDT). The established reaction protocol^40^ of simply mixing the monomers in DMF at room temperature proved to be applicable, yielding the corresponding TCC-polymers poly­(1,4-BDT/BQA) (**P1**), poly­(1,3-BDT/BQA) (**P2**) and poly­(1,2-BDT/BQA) (**P3**). The final polymer products were isolated by consecutive precipitations in MeOH/H_2_O 3:2 and pure H_2_O to remove low-molecular-weight fractions characteristic of polyaddition reactions and the polymers were thoroughly characterized spectroscopically and chromatographically (Figures S1–S6).

As anticipated, the reaction mixtures with 1,3-BDT and 1,4-BDT decolorized within about 5 s, indicating rapid and nearly quantitative consumption of BQA. The polymerization mixture with 1,2-BDT, however, retained a reddish color for hours, suggesting incomplete BQA conversion, but proceeded to decolorize completely after addition of excess ethanethiol. This observation could be attributed to the tendency of 1,2-BDT to dimerize under oxidative conditions,^50^ depleting monomers, and to steric hindrance from the 1,2-substitution pattern, which likely slows polymer growth. For **P1** and **P2**, size-exclusion chromatography (SEC) confirmed the formation of TCC-polymers and indicated the end of the polymerization after about 15 min, as molecular weights did not increase further after that (Figure S7). Polymerization with 1,4-BDT yielded the highest molecular weight TCC-polymer **P1** with *M*_*w*,app_ = 34,000 g mol^–1^ (*Đ* = 2.3), while 1,3-BDT resulted in polymer **P2** with *M*_*w*,app_ = 14,000 g mol^–1^ (*Đ* = 1.7). In contrast, 1,2-BDT led only to low molecular weight polymer **P3** with *M*_*w*,app_ = 2500 g mol^–1^ (*Đ* = 1.3). As indicated by the slow decolorization of the reaction mixture, SEC further underpinned the limited efficacy of the *ortho*-isomer. The expected 1:1 incorporation-ratio of AA/BB monomers was confirmed by NMR for all polymer products, indicating that the polyaddition reaction proceeded according to the established mechanism for TCC-polymerizations^40^ and suggesting within the error of the method no dramatic alternative growth pathways e.g. via disulfide bonds in the backbone. Furthermore, the latter could be excluded by SEC, where the addition of tributyl phosphine, which cleaves disulfides, showed no significant effects on the molecular weight distribution, as exemplified with **P1** (Figure S8).

It is well-known that backbone symmetry and conformational flexibility have an effect on chain segment mobility and thus influence the glass transition temperature (*T*_g_) of a polymer.^51,52^ Dynamic scanning calorimetry (DSC) analysis provided high *T*_g_ values for **P1** and **P2** with 153 and 142 °C, respectively (Figure S9). The low molecular weight **P3** has been omitted from analysis. As expected, the benzenedithiol polymers **P1** and **P2** exhibited higher *T*_g_ values compared to previously described TCC-polymers from conformationally more flexible monomers, such as 1,4-benzenedimethanedithiol (1,4-BMT) or 2,2’-(ethylenedioxy)­diethanethiol (EDET) with *T*_g_ values of 121 and 68 °C, respectively.^39^ Those TCC-polymers (poly­(1,4-BMT/BQA) (**P4**) and poly­(EDET/BQA) (**P5**)) were also included as controls to study the effect on paper strengthening by more flexible TCC-polymers.

With this set of TCC-polymers offering functionalities with strong interactions, their efficacy as reinforcement for cellulose was investigated. Initial impregnation experiments were conducted, using commercially available filter paper (Macherey-Nagel MN 616), either by dip-coating in a solution of preformed TCC-polymer or by sequentially preloading the paper with BQA, followed by addition of dithiol. Both approaches resulted in TCC-polymer deposition. Direct deposition of preformed polymer yielded only surface deposition, due to the immediate precipitation of the highly adhesive TCC-polymers (Figure S10).^27^ Similarly, BQA-preloaded paper strips produced spotty, inhomogeneous coatings with substantial amounts of unreacted BQA, likely caused by strong local concentration gradients within the pores (Figure 2).

**Figure 2:**
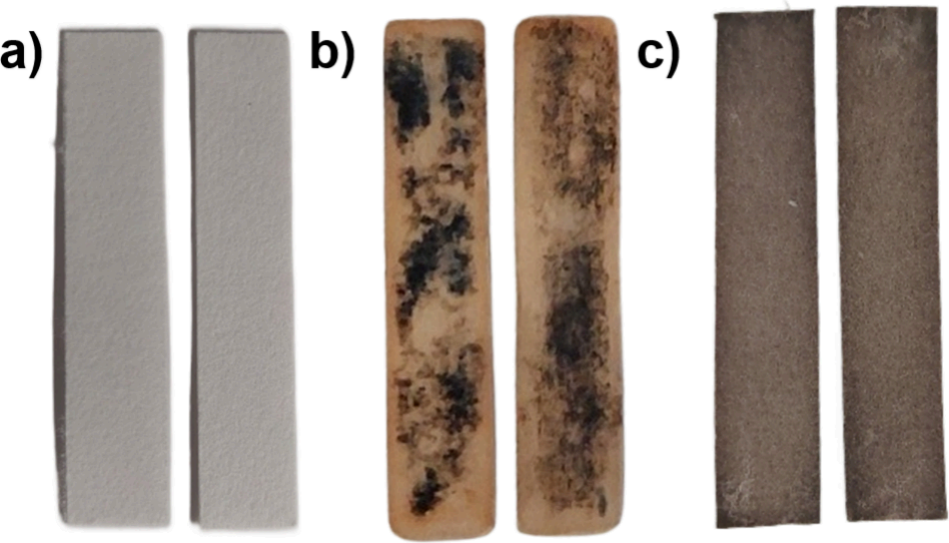
Optical comparison of filter paper samples. Pristine filter paper (a), inhomogeneous and spotty coatings obtained from BQA-preloaded paper strips after dithiol addition (b), and homogeneous coating produced using the InSPA methodology, showing the beige-brown color characteristic for TCC-polymers (c).

The in-sheet polymerization and adhesion (InSPA) strategy avoids those difficulties and facilitates uniform TCC-polymer distribution by forcing the penetration of the coating throughout the paper network. The interactions of TCC-polymers with cellulose are likely governed by reversible H-bonding and van der Waals contacts, as supported by studies on catechin-cellulose binding and peptide-based cellulose-binding modules of glycoside hydrolases.^53,54^ To achieve homogeneous dispersion of highly interactive TCC-polymers within the cellulose matrix, the polymerization rate had to be significantly reduced. This was accomplished by lowering the monomer solution temperature, thereby allowing unreacted monomers sufficient time to infiltrate the fiber network. Cooling was conveniently performed using an acetonitrile/liquid nitrogen slush bath at −43 °C, a temperature still above the freezing point of DMF (−61 °C). Reaction kinetics monitored by UV/vis-spectroscopy at 377.5 nm revealed a delay in reaching 90% conversion from ∼6 s at 20 °C to ∼23 s at −43 °C, thus providing sufficient time for the dip-coating procedure (Figure S11). The polymerization thus takes place within the pore system of the paper, progressively increasing molecular weight and adhesiveness of the TCC-polymers, causing the polymers to deposit onto the cellulose fibers (cf. Figure 1).

A sequential approach proved to be most practical, wherein each monomer was individually dissolved in DMF, cooled to −43 °C, and subsequently combined, prior to briefly immersing the precooled paper strips in the monomer mixture (5 per batch, 5 cm × 1 cm). After ∼1 s, the paper was removed and excess solution was allowed to drain off, minimizing residual surface droplets. The fully soaked paper strips were left to polymerize in a DMF-atmosphere at ambient temperature. The characteristic coloration faded completely over seconds to minutes, as the samples gradually reached room temperature, indicating reaction progress. However, samples were left undisturbed for 15 min to ensure complete polymerization. The samples were subsequently dried for 1 h at 90 °C to restore capillary forces by evaporating residual solvent and reopening the voids between the fibers to enable fluid uptake during the subsequent dip-coating cycle. This enables the samples to undergo further InSPA loading cycles until the desired polymer content is reached. The resulting sample sets are hereafter denoted as **Px@FP** ^**y**^, where Px describes the polymer and y indicates the number of soaking cycles. Each of the thiol isomers was subjected to the procedure, yielding optically homogeneous beige to brown, polymer-infused paper samples (**P1@FP** ^**5**^, **P2@FP** ^**5**^ and **P3@FP** ^**5**^) (Figure 2c).

To evaluate the loading process, the polymer uptake was followed by gravimetry (Figure 3a). Mass uptake of the sheets could be reproducible tailored and increased by about 7 wt.-% for each InSPA loading cycle. An approximately constant increase was observed over the first five cycles for all three isomeric TCC-polymers. For **P1**, further loading was carried out up to ten cycles (**P1@FP** ^**10**^). The initial, rather linear increase was followed by a smaller increase that is found after 5 cycles, which might be attributable to the saturation of the cellulose fibers with TCC-polymers. After ten cycles, 48 wt.-% of **P1** had been adsorbed onto the paper with the possibility to be increased by additional loading cycles. Although the successive monomer-loading/polymerization protocol is rather complex, the InSPA process proved reproducible, yielding uniformly coated samples. This was demonstrated by generating ten individual **P1@FP** ^**10**^ samples, which after 10 cycles exhibited an average mass of 273 ± 11 mg, corresponding to a standard deviation of only 4%. After the last loading cycle and drying step, the finished paper samples were evaluated for their mechanical properties (vide infra) and subsequently washed with the TCC-polymer nonsolvent DCM to extract monomers and oligomers. The polymer loading of the papers decreased on average only by marginal 1–2 wt.-%, indicating the coating has a dominantly higher molecular weight nature. In a second step, a DMF wash resulted in the extraction of practically all TCC-polymer, as evidenced by gravimetry, producing only white paper strips (cf. section 3.4). The complete removal of the polymer from the paper was consistent with the noncovalent nature of the coating. SEC analyses of the DMF-extracts show the formation of polymers with broad molecular weight distributions and molecular weights fractions that reach up to 50 × 10^3^ and 30 × 10^3^ g mol^–1^ for **P1** and **P2**, respectively (Figure 3b). **P3** was found to be dominated by oligomers, as could be expected from the solution experiments. Intriguingly, the samples proved impervious to washing with water, as discussed in more detail in section 3.4 together with the recycling experiments.

**Figure 3:**
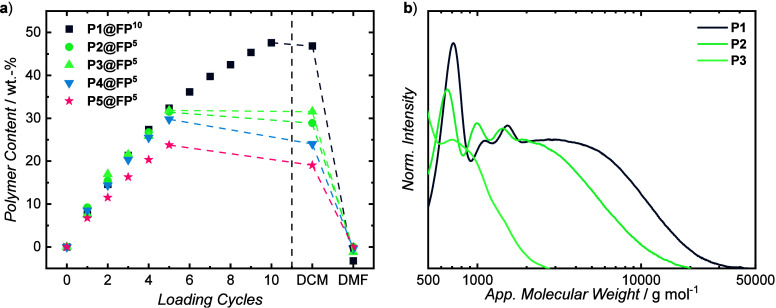
Preparation of TCC-infused paper samples following the InSPA methodology. Gravimetric analysis of the preparation of **P1@FP** **^10^**, **P2@FP** **^5^**, **P3@FP** **^5^**, **P4@FP^5^**, and **P5@FP^5^** over several soaking cycles as well as after washing with DCM and DMF (a). SEC-traces of the respective polymers extracted from **P1@FP** **^10^**, **P2@FP^5^**, and **P3@FP^5^** using DMF (b) [Conditions: 12.8 mg mL^–1^ dithiol, 20.8 mg mL^–1^ BQA in DMF, −43 °C-RT, 15 min].

The comparison between the DMF-extract of **P1@FP** ^**5**^ and the polymerization product formed in the dip-coating supernatant supported the anticipated InSPA mechanism outlined in Figure 1, as the polymer fraction formed in the pore network clearly exhibited a significantly lower molecular weight compared to the product formed freely in the supernatant (Figure S12). These findings are consistent with the progressive formation of interaction capabilities throughout the polymerization and further confirm that TCC-polymers begin associating with cellulose fibers early in the process. Moreover, the deposition of the TCC-polymer onto cellulose may slightly hinder/slow further chain growth, although polymerization remains ongoing.^51^ This could potentially also account for the presence of oligomers in **P1@FP** and **P2@FP**.

### 3.2 Mechanical and Microscopic Analysis of the Paper Samples

The impact of the InSPA coating was assessed in an initial screening using a nonstandardized three-point bending setup, which revealed a significant influence of the different TCC-polymers on the mechanical properties of the paper composites. The tests resulted in qualitative data in reference to pristine filter paper only, which nevertheless allowed for comparison between the different polymers. For these measurements, the strips were placed on a two-point support and bent at middle position perpendicular to the surface using a metal stamp (adapted from ISO 5628).^55^ The force encountered by the stamp was recorded, fitted and averaged over each sample set (Figure 4a, Table S1, and Figures S13–S19).

**Figure 4:**
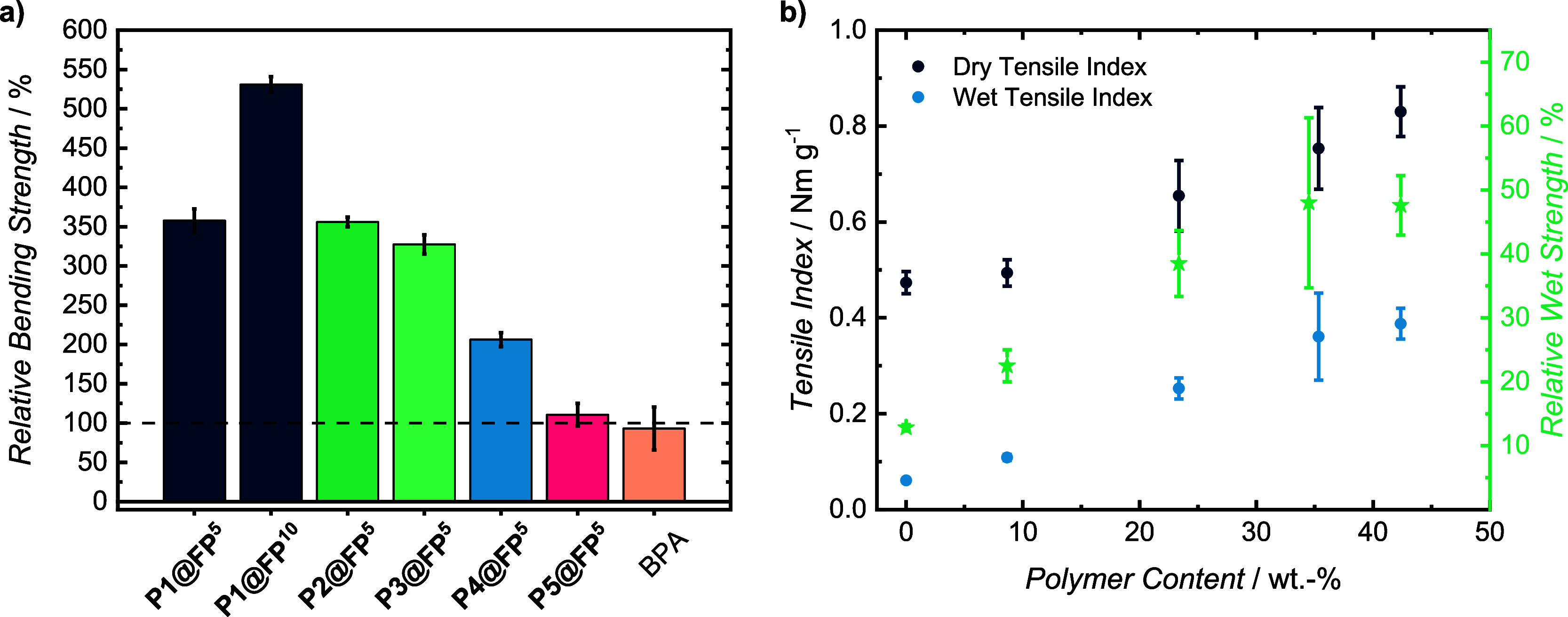
Influence of polymer type and loading on the mechanical strengthening of paper samples. Qualitative comparison of the bending strength of samples coated with various TCC-polymers or model compounds. The bending strength of the pristine filter paper reference is shown as a dashed line at 100% (a). Quantitative tensile index values obtained from samples of **P1@FP** with varying polymer content. The relative wet strength corresponds to the ratio between the wet and dry tensile indices. The value for **P1@FP^7^** has been slightly offset for clarity (b) [Conditions: 12.8 mg mL^–1^ dithiol, 20.8 mg mL^–1^ BQA in DMF, −43 °C-RT, 15 min].

After five soaking cycles that resulted in approximately 28–32 wt.-% polymer loading, the average stiffness of the samples increased significantly by three- to 4-fold. As the samples were prepared under identical conditions and show similar loadings within the experimental error, the chemical nature of the coatings is presumed to strongly dominate the effect on paper stiffness. A quantitative analysis giving absolute bending stiffness values was not undertaken, as the available setup did not conform to ISO standards, and minor variations in thickness cannot be fully excluded as contributing factors. While **P1@FP** ^**5**^ and **P2@FP^5^** samples show the highest strengthening to 356% ± 6% and 358% ± 15% their original strength, **P3@FP^5^** realized slightly less strengthening to 327% ± 12%. Increasing the polymer amount to 48 wt.-% by further soaking cycles resulted in **P1@FP** **^10^**, which displayed a relative strength of 531% ± 10%. This proves that the polymer coating transfers rigidity to the fiber network, making the paper stiffer, which benefits from an increase in polymer fraction. Control experiments using the low molecular weight analogue bisphenol A (BPA) were conducted following the InSPA coating protocol. The procedure achieved a 34 wt.-% loading but showed no significant stiffness enhancement (93% ± 27%), indicating that mechanical reinforcement of paper requires the presence of TCC-polymers.

Interestingly, the remarkable paper stiffening effects were rather similar across the TCC-polymers **P1**, **P2**, and **P3**, despite their differing *T*_g_. To further investigate the role of TCC-polymer properties on mechanical paper reinforcement, the InSPA process was replicated with 1,4-BMT and EDET to produce **P4** and **P5**, which exhibit significantly lower *T*_g_ values of 121 and 68 °C, respectively. When subjected to identical loading conditions, both paper sets showed a linear increase in polymer content, reaching 30 wt.-% for **P4@FP** ^**5**^ and 24 wt.-% for **P5@FP** ^**5**^. The mechanical properties of the sets follow the *T*_g_ values of the respective polymers, with **P4@FP** ^**5**^ achieving a less pronounced reinforcement to 206% ± 9% its original strength and **P5@FP** ^**5**^ barely increasing stiffness at all, reaching a value of only 110% ± 14%. Although the data is qualitative, the influence of the polymer backbone structure can be deduced, with fully aromatic BDT monomers yielding significant stiffening, benzylic BMT monomers providing moderate reinforcement, and flexible EDET monomers having minimal impact. These results suggest that the superior reinforcing ability of **P1** and **P2** arises from their rigid molecular architecture that leads to high *T*_g_.

In subsequent studies, the most promising isomer 1,4-BDT was selected, as it was found to give the highest molecular weight TCC-polymers under both the solution polymerization and InSPA conditions. To complement the initial bending measurements, quantitative tensile tests were conducted on **P1**-strengthened paper samples. Interestingly, the increasing polymer content correlates well with a rise in both dry and wet strength, which apparently was not leveling off even at high additive contents of 42 wt.-% **P1**.

In addition to a reference set of pristine filter paper, five sample sets, each comprised of ten 12 cm × 1.5 cm strips, were prepared using the InSPA process for 1, 4, 7, and 10 cycles under standardized conditions to provide **P1@FP** ^**1**^, **P1@FP** ^**4**^, **P1@FP** ^**7**^ and **P1@FP** ^**10**^. For any given set, half of the paper strips were fully soaked in water for wet strength testing and all samples were analyzed mechanically in accordance with ISO 1924-2 (Figure 4b, Table S2, and Figures S20–S24).^56^ The pristine filter paper sample displays a tensile index of 0.47 ± 0.02 Nm g^–1^ in the dry state and 0.06 ± 0.00 Nm g^–1^ in the wet state, which corresponds to 13% relative wet strength as the ratio between wet index and dry index.^65^ The decreased strength in the wet state was to be expected since water swells the **FP** and interrupts the intermolecular bonds between the individual fibrils, lowering network cohesion.^66^ However, since a relative wet strength of >15% is still usually considered high, a value of 13% hints at the incorporation of small amounts of wet strength agent into MN 616, as confirmed by the manufacturer. Upon coating to 42 wt.-% **P1**, the dry tensile index reached up to 0.82 ± 0.05 Nm g^–1^, constituting a doubling. More impressively, the wet index increased to 0.39 ± 0.03 Nm g^–1^, achieving 7 times the original wet index. The relative wet strength increases rather linearly up to 48% at 35 wt.-% **P1**. At higher TCC-polymer contents, the wet strengthening effect levels off at ∼47% after seven InSPA cycles and **P1** contents up to 42 wt.-%. Remarkably, the TCC-polymer coating therefore not only strengthens the paper in its dry state but also acts as a highly effective wet strength agent. The leveling-off of the relative wet strength at high polymer loadings is attributed to fiber surface saturation and is has been observed for other wet strength additives.^24^

Moreover, a steady decrease in the wetting rate was observed for samples with higher TCC-polymer loading, suggesting an increase in surface hydrophobicity and reduced water intercalation. To investigate, the samples used to determine dry tensile strength were further subjected to contact angle measurements with water (Figure S25). The behavior of the water contact angle with polymer loading mimicked the behavior of the relative wet strength. While no contact angle could be determined for **FP** due to immediate infiltration of the droplet, the contact angle increased continuously with polymer loading and leveled off at TCC-polymer contents of ≥35 wt.-%. **P1@FP^10^** expectedly displayed the highest contact angle of 126.8° ± 4.1°, with no infiltration of water observed over several minutes. Presumably, the hydrophobic nature of the **P1** coating reduces the wettability of the fiber network and thereby maintains the reinforcing effect of the rigid coating under wet conditions. This mechanism resembles the behavior of internal sizing agents such as the alkyl ketene dimer (AKD) and alkenyl succinic anhydride (ASA), which hydrophobize cellulose fibers, preventing water penetration and fiber swelling.^19^ The pronounced hydrophobicity of the surface is likely facilitated by entrapped air within the paper pore-structure.^57^ As both hydrophobicity and relative wet strength increase with higher **P1** content, it might be reasonable to assume that these properties level off once a sufficient TCC-polymer coverage is reached, which appears to occur at ∼35 wt.-% **P1** within the paper samples.

To further characterize the polymer coating on the paper fibers, **P1@FP^10^** samples were analyzed using microscopy and spectroscopy. Following multiple InSPA cycles, the initially white **FP** sheets developed a uniformly beige/brown coloration, indicative of the formation and homogeneous distribution of the **P1** TCC-polymers across the fiber surfaces (cf. Figure 2). Washing with DCM did not noticeably change the paper color, whereas treatment with DMF restored the samples to a visually white appearance. The mechanical reinforcement observed in paper samples after multiple InSPA cycles was reversed, resulting in the regeneration of the initial soft and flexible paper properties. To examine the structural changes throughout the modification process, the samples were analyzed at various stages using scanning electron microscopy (SEM). Figure 5a shows SEM-micrographs of the paper sample before (i) and after (ii) being infused with 48 wt.-% **P1** as well as after washing with DCM (iii) and after the final extraction of the TCC-polymers with DMF (iv).

**Figure 5:**
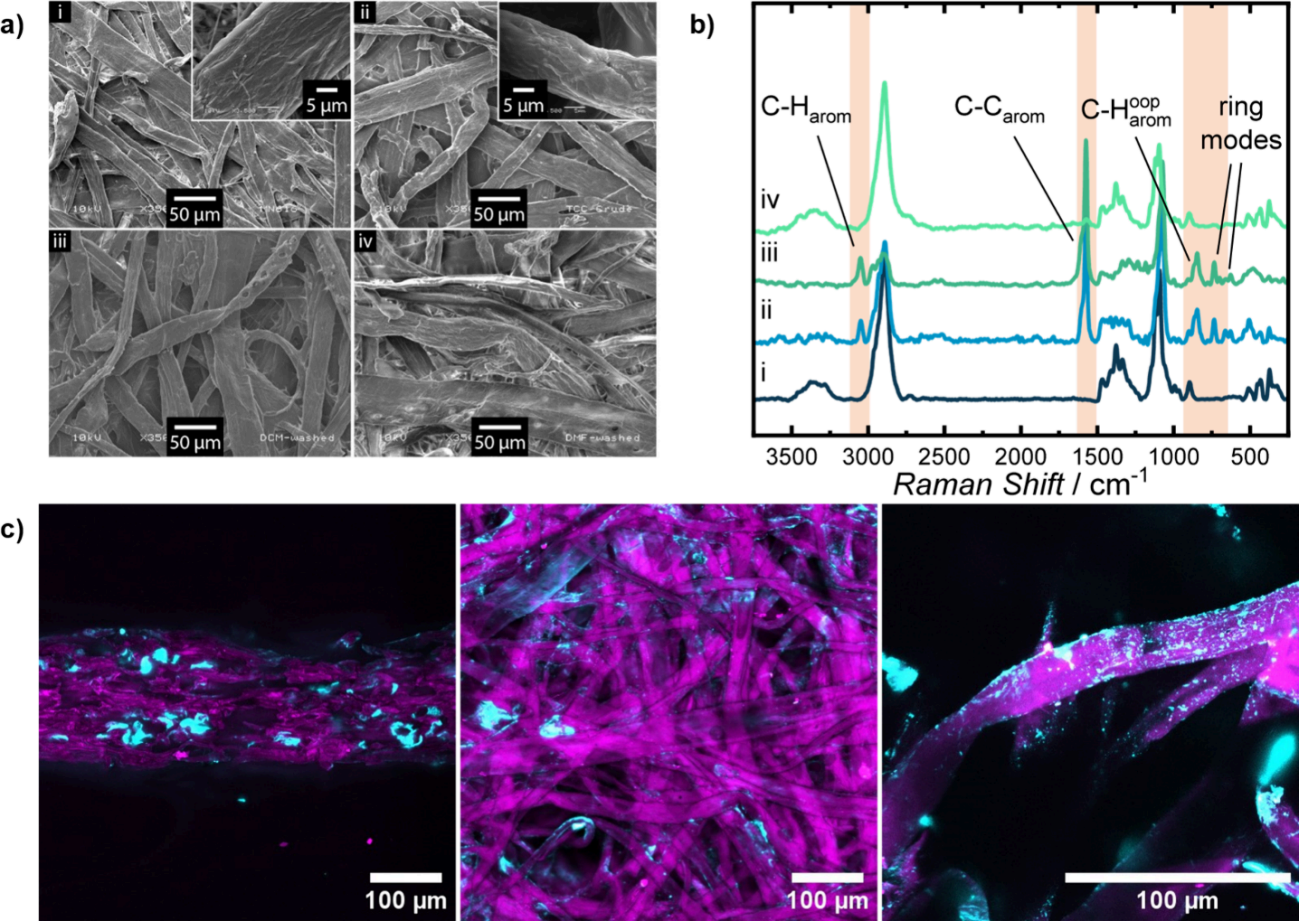
Microscopic and spectroscopic analyses of TCC-infused paper samples at different stages of preparation. SEM micrographs of pristine MN 616 filter paper **FP** (i) and **P1@FP** ^**10**^ directly after preparation (ii) as well as after washing with DCM (iii) and DMF (iv) (a). Raman-spectra of fibers of **P1@FP** ^**10**^ in different stages of preparation, analogous to the SEM micrographs (b). CLSM-micrographs of the paper cross section (left), maximum projection of the top-down view (middle), and the magnification of an individual fiber in a top-down view (right) of P1*@FP ^**10**^, with TCC-polymer displayed in pink and native cellulose displayed in cyan (c).

It is noteworthy that even an extremely high TCC-polymer content still preserves the structural integrity of the fibrous cellulose network, which remains intact, with fibers and pores being distinguishable across multiple surface layers. The fiber surface morphology appears intact and mostly unchanged despite the high TCC-polymer loading, as confirmed by comparative imaging (Figure 5a insets i and ii), and no significant polymer agglomeration or pore occlusion was observed. This evidenced a uniform polymer distribution along the cellulose fibers. However, given the inherent limitations of micrographs in terms of chemical specificity, spectroscopic analysis was employed to further elucidate TCC-polymer distribution throughout the sample.

As the features of the paper are sufficiently large, Raman-microscopy could be used to provide insight into the chemical composition of the fiber surface, even though samples proved to be challenging due to high autofluorescence, which might be caused by trace lignin impurities.^58^ Raman-spectra, analogous to the SEM-micrographs, are depicted in Figure 5b. The Raman-spectrum of pure **FP** exhibits characteristic cellulose features with prominent vibration bands at 1092 and 1118 cm^–1^, corresponding to (C–C) and (C–O–C) stretching vibrations of the cellulose backbone, respectively.^59^ Additional vibration bands between 1220–1500 cm^–1^ were attributed to (H–C–C), (H–C–O), and (H–O–C) bending modes, while the (C–H) stretching vibration appeared at 2891 cm^–1^. Notably, no spectral features indicative of aromatic structures could be detected in **FP**. The Raman-spectrum of the crude **P1@FP** ^**10**^ paper sample exhibits new absorption bands characteristic to the aromatic 1,4-BDT and BQA units. The two distinct bands at 1572 and 3048 cm^–1^ correspond to aromatic C–C and C–H stretching vibrations, respectively.^60^ The weaker bands at 737 and 629 cm^–1^ correspond to aromatic ring modes as observed in the 1,4-BDT monomer (Figure S26) and the band at 851 cm^–1^ is tentatively assigned to C–H out-of-plane vibrations of the catechol unit. The absence of strong S–H stretching bands near 2554 cm^–1^ and conjugated C=O stretching bands in the region between 1600–1700 cm^–1^ indicates minimal presence of unreacted monomers or oligomers and suggests high polymerization efficiency. This was supported by the close similarity between the spectra of crude and DCM-washed **P1@FP** ^**10**^ paper samples, as low molecular weight compounds would be extracted. The spectrum of the DMF-washed sample lacks the characteristic **P1** bands and is almost identical to the initial **FP** spectrum, proving the complete removability of the TCC-polymer from the paper and confirming the noncovalent character of the TCC-polymer coating. The uniformity of the **P1@FP** ^**10**^ paper sample was assessed by sectioning the coated paper network into smaller pieces and measuring at the cross section. Spectral comparison of coated fibers at the surface and within the bulk of the paper revealed strong agreement, indicating homogeneous distribution of the **P1** coating throughout the sample (Figure S27).

To evaluate the three-dimensional distribution of **P1** within the paper matrix and the cellulose fibers, a fluorescently labeled sample P1*@FP^10^ was prepared by replacing 1 mol-% of 1,4-BDT with a fluorescent dithiol during the InSPA process. Residual uncoated cellulose fibers were stained with Calcofluor White, and the sample was imaged using confocal laser scanning microscopy (CLSM), enabling spatially resolved visualization of P1* localization (Figure 5c). Staining with Calcofluor White was proven to be selective for cellulose and did not lead to staining of the polymer (Figure S28), which allowed for the distinction between coated and uncoated areas. As expected, the fibers were homogeneously coated with P1*, with no aggregates of TCC-polymer observed within the paper pore system or at the surface of the paper sheet. Intriguingly, only small patches of noncoated cellulose fiber surfaces were detectable, while the remaining fibers were covered by the TCC-polymer, thereby preventing Calcofluor White-binding. These observations confirm the excellent homogeneity of the TCC-polymer coating formed by the InSPA process and support the previous assumption that saturation of the cellulose fiber with TCC-polymer restricts the achievable effects of enhancing hydrophobicity and relative wet strength. Interestingly, the InSPA coating enables the TCC-polymer to penetrate into the fiber lumen as well, where it is apparently also homogeneously distributed. This was supported by the magnified cross section in Figure S29, which shows polymer signals within the fiber lumina rather than solely at the fiber surface. The penetration into the fibers can be rationalized by the high swelling capability of DMF amplified by the high mobility of the small molecule reactants provided in the InSPA coating methodology. A dispersion of TCC-polymers throughout the fiber lumen would similarly explain the saturation effects at high TCC-polymer loadings that have been observed gravimetrically. Thus, the primary effect of the TCC-polymer additive appears to result from modification of the cellulose fiber bulk and interfaces, rather than from encapsulation of fibers by a dense TCC-polymer coating. It could be speculated, that additional InSPA coating steps, leading to a thickening of the TCC-polymer layer, would yield diminishing returns in terms of modifying fiber mechanics.

### 3.3 Paper Membrane Filtration in Organo-/Water Filtration Application

The InSPA process preserved the continuous pore system in the filter paper, while enhancing both wet strength and hydrophobicity. These three material parameters are often critical for technical filtration applications, making the enhanced paper interesting for membrane separation applications. Microscopic analysis and contact angle measurements confirmed that **P1@FP^10^** exhibits promising material characteristics, prompting an investigation into how TCC-reinforcement influences selective liquid separation. Notably, during contact angle measurements **P1@FP^10^** was immediately wetted and infiltrated by cyclohexane, whereas water infiltration was completely suppressed over the course of the experiment (Figure 6a). In contrast, untreated, dry **FP** absorbed both nonpolar organic solvents and aqueous solutions (Figure S30). However, once wetted with water, **FP** exhibited oleophobic properties,^61^ effectively blocking the passage of nonpolar liquids. The selectivity of **P1@FP^10^** was utilized in initial model experiments, separating cyclohexane from water. In a dual-membrane setup (Figure 6b), a **P1@FP^10^**-membrane was placed on the left for hydrophobe extraction, while a water-prewetted **FP**-membrane on the right enabled water separation. To allow for visual distinction of the two liquids, the organic cyclohexane phase was stained red with Sudan IV and the aqueous phase was dyed blue with methylene blue. Over the course of the experiment, effective separation of the mixture was observed, with water only percolating through **FP** and the organic solvent only percolating through the TCC-membrane. These findings were reproduced in a vertical setup, where vigorous stirring allowed the cyclohexane phase to repeatedly contact the **P1@FP^10^**-membrane and percolate, with the water being retained above the filter (Figure S31).

**Figure 6:**
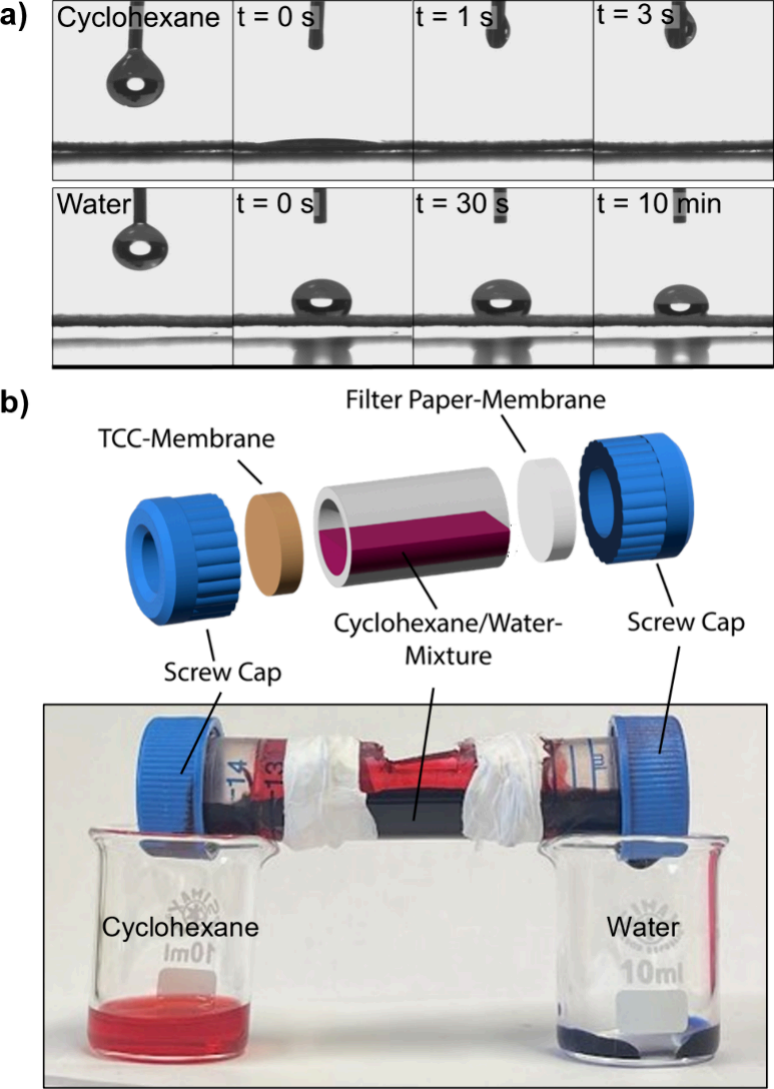
Assessment of hydrophobic **P1@FP^10^** as a selective filter membrane for nonpolar liquids. Contact angle measurements of cyclohexane and water on **P1@FP^10^** (a) and filtration setup for cyclohexane/water (1:1) separation (b).

These separation experiments served as initial proof of concept, with the application leveraging the combination of the hydrophobization, the unobstructed pore system and the enhanced wet strength of the paper to provide mechanically robust membranes.

### 3.4 Recycling of Polymer-Enhanced Composite Papers

Integrated polymer-cellulose hybrid materials frequently enable access to advanced functions but often suffer from poor end-of-life disassemblability, limiting the overall benefit of sustainable hybrid materials. The recyclability of reinforced filter paper samples was investigated, showcasing the noncovalent nature of InSPA wet strength coatings. Gravimetric and spectroscopic analyses confirmed that DMF washing efficiently removed the TCC-polymer from e.g. **P1@FP^10^** samples. This ease of separation contrasts with many conventional wet strength additives, which are often covalently bound to the fibers and are therefore more difficult to remove.^21,31^ However, organic solvent extraction procedures do not reflect industrial paper recycling processes, which rely on aqueous solutions and mechanical treatments to remove contaminants from the fibers before repulping.^62^ To investigate the possibility and practicability of a removal of the TCC-coatings in aqueous systems, two samples of **P1@FP^10^** were extracted with 1 M HCl and 1.25 M NaOH, respectively. No obvious change of the paper sample or the washing solution was found for the acidic treatment, whereas the paper washed under basic conditions lost its brownish color, indicating the removal of the TCC-polymer. Moreover, the supernatant solution developed a dark green color, indicative of charge-transfer-complexes between catechols and quinones, with the latter potentially (re)­formed by air oxidation under alkaline conditions (Figure S32).^63^ This assumption was supported by the observation of a similar discoloration in BQA solutions upon treatment with NaOH (Figure S33). Therefore, in a second extraction of a **P1@FP^10^** sample with aqueous NaOH solution, an excess of ascorbic acid (Asc) was added to suppress the formation of *ortho*-quinones. The second extraction produced a clean white paper sample along with a slightly discolored supernatant, likely due to degradation of the ascorbic acid (Figure S34).^64^ Aqueous NaOH solutions are known to actively disrupt cellulose fiber integrity^65^ and deprotonate phenolic hydroxy groups. It can be hypothesized, that these processes contribute to breaking crucial H-bonds, enabling cellulose fiber swelling and subsequent dissolution of TCC-polymers as polyanions. After extraction, the paper samples were thoroughly washed with water, carefully dried, and analyzed by Raman-spectroscopy. No TCC-polymer signals were detected within the error of the method, indicating practically complete removal of TCC-polymer additives through aqueous recycling (Figure S35). As additional evidence for the effective removal of TCC-polymers, gravimetrical analysis was performed by comparing the dry mass of paper strips before and after solvent treatment. Treatment with either DMF or 0.22 M Asc in 1.25 M NaOH resulted in nearly quantitative polymer removal within the error of the method (Figure S36, Table S3), confirming the high efficacy of both solvent systems. Remarkably, the solvents seem to be able to extract polymers not only from the fiber surface but also from within the fiber lumen itself. In contrast, samples washed with deionized water showed only minor weight loss.

After confirming the removal of the coating using both organic and alkaline aqueous solvent systems, recycling tests of **P1@FP^10^** were conducted using a modified protocol based on the work by Pfennich et al.^49^ In this method, paper samples are disintegrated using minimal energy input and the resulting pulp is reformed into new sheets through fine-sieve dewatering. The extent of disintegration was quantified by a dispersibility score (DS) ranging from 0 (no disintegration) to 10 (complete disintegration), taking the size and amount of residual paper fragments into account. In three separate experiments, the recyclability of samples of **P1@FP^10^** and a pristine **FP** reference were investigated following polymer extraction with either water, DMF or aqueous NaOH/Asc. The extracted sample sets were disintegrated for 20 min at 900 rpm using an overhead stirrer equipped with a propeller-type agitator. The resulting pulps were dewatered through a fine sieve and subsequently analyzed visually (Table S4).

Notably, the TCC-polymer-rich and strongly hydrophobic paper sample **P1@FP^10^** was almost completely disintegrated (DS = 8–9) after extraction with aqueous NaOH/Asc, while extraction using pure water proved entirely ineffective (DS of 0). The use of aqueous solvent renders the process more compatible with technical repulping strategies and exemplifies the principles of green chemistry for the recyclability of TCC-strengthened materials. Interestingly, aqueous NaOH/Asc was shown to be even more effective than the DMF extraction, which yielded lower disintegration scores of DS = 5–6. In contrast, the unmodified **FP** samples already exhibited moderate disintegration after water extraction, with a DS of 5. Treatments with either DMF or aqueous NaOH/Asc resulted in similar or slightly improved scores compared to **P1@FP^10^** (DS = 6–7 for DMF; DS = 8–9 for NaOH/Asc). The improved repulping performance of NaOH is most likely due to enhanced fiber swelling and partial loosening of the fiber network under alkaline conditions, as supported by mechanical tests of pristine filter paper before and after the washing (Figures S37–S40, Table S5). Notably, only NaOH/Asc washing caused a moderate reduction in tensile strength in both dry and wet states, consistent with the slightly higher disintegration scores of these samples.

While further optimization of conditions may be possible, the removal of the TCC-polymer – particularly by aqueous NaOH/Asc solutions – has been successfully translated into the formation of new paper sheets from the highly strengthened **P1@FP^10^** samples, despite the fact **P1@FP^10^** remains completely unaffected by mechanical agitation in pure water. The recyclability demonstrated in the experiments underscores the potential for reintegration of fibers, treated with the noncovalent TCC-polymer dry/wet strength agent, into the materials cycle after use.

## 4 Conclusions

The *in-sheet polymerization and adhesion* (InSPA) methodology was established to noncovalently reinforce premade paper sheets with high-*T*_g_ polymers featuring highly adhesive thiol-catechol connectivities (TCCs). The approach enabled homogeneous infiltration of reactive, nonadhesive monomers at lower temperatures, allowing penetration down to the cellulose fiber lumen. A clean room-temperature Michael-type polyaddition subsequently formed TCC-polymers, which gradually increased adhesiveness and deposited noncovalently onto the cellulose network, resulting in a uniform coating and significantly enhancing paper strength under both dry and wet conditions. Building on the successful establishment of the InSPA process for wet strength reinforcement, future studies will aim to provide a more quantitative understanding of the intermolecular interactions between TCC-polymers and cellulose fibers. A systematic investigation of untreated fiber networks might further clarify the fundamental mechanisms of reinforcement and broaden the potential applicability across diverse fiber systems. While direct spectroscopic evidence of the interaction mechanism remains difficult to obtain, as the cellulose-polymer interface represents only a minor fraction of the bulk TCC-material, ongoing efforts using thin-layer coatings or single-molecule force measurements and computational simulations might reveal deeper insights and will be reported elsewhere.

A small library of three TCC-polymers was prepared via the InSPA process, using bisquinone A and the three isomeric dithiols 1,4-, 1,3- and 1,2-benzenedithiol (BDT). The TCC-polymer loading onto filter paper (**FP**) could be adjusted up to 48 wt.-% without obstruction the pore system, while measurable effects were already observed at lower loadings. These polymer fractions fall within the range of paper-based composites, a class of materials commonly used in specialized applications such as construction^66^ or multilayer beverage cartons with typical fiber-to-polymer ratios around ∼75:20.^67^ This illustrates that the material compositions accessible with the InSPA approach might be potentially relevant for industrial use. The poly­(1,4-BDT/BQA) (**P1**) proved most effective, achieving reinforced filter paper (**P1@FP** ^**10**^) with double the dry tensile index and a remarkable 650% improvement in wet tensile index, to give a relative wet strength of 47%. Although InSPA shows strong potential for producing durable and hydrophobic paper-polymer-composites and occupies a specific niche for coatings with highly adhesive polymers, the current reliance on repeated dip coating in DMF at low temperatures limits scalability. While a scale-up from 8 to 55 mL batches was accomplished, the method remains a proof of principle with further room for improvements, likely requiring more advanced setups (e.g., cooled size presses) and a transition to more benign solvents such as *N*-butyl-2-pyrrolidone or ultimately aqueous dispersions. In the composites, the polymer was confined within and along the fibers, preserving the pore structure and enabling solvent/water filtration tests in which selective cyclohexane percolation was observed. Despite the high polymer loading and interlaced composite nature, **P1@FP** ^**10**^ was fully recyclable, with quantitative removal of TCC-polymer by an aqueous solution of NaOH and ascorbate. Notably, the repulpability of **P1@FP** ^**10**^ after TCC-polymer removal, matched that of pristine filter paper in recycling demonstrator tests. In the future, circularity of the material system might be enhanced by reusing TCC-polymers in secondary applications, such as adhesives or coatings. Depolymerization of TCC-polymers via reversible Michael-addition through selective sulfide cleavage might offer an alternative pathway, though it would require significant optimization and has not previously been described.^68–70^ The InSPA methodology for paper reinforcement thus offers a promising route to recyclable filter membranes with tunable mechanical properties and hydrophobicity.

## Supplementary Material



## Notes

Characterization of the individual polymers (TGA, DSC, ^1^H NMR, SEC, FTIR, kinetic studies); results of the qualitative bending measurements and quantitative tensile tests; additional characterization of polymer-infused paper samples (photographs, contact angle measurements, supplemental Raman-spectra and CLSM-micrographs); supplemental filtration data; results of recycling experiments (PDF)
