# Supplementary material for: Strength from Within: Reversible Reinforcement of Paper through In-Sheet Formation of Thiol-Catechol Polymers
Source: ACS Appl Mater Interfaces. 2025 Oct 27;17(44):61164–76. doi: 10.1021/acsami.5c13781 (PMC12598705; doi:10.1021/acsami.5c13781)
Supplement: Supplementary file 1 [file am5c13781_si_001.pdf]

Supporting Information:

Strength from Within: Reversible Reinforcement of Paper through In-Sheet Formation of Thiol-Catechol-Polymers

Lukas D. Bangert‡,[a], Nicole Kirchner‡,[b], Ching-Yi Choi[a], Markus Biesalski[b],, Hans G. Börner[a],**

[a] L. D. Bangert, C.-Y. Choi, H. G. Börner

Humboldt-Universität zu Berlin, Department of Chemistry, Laboratory for Organic Synthesis of Functional Systems, Brook-Taylor-Str. 2, 12489 Berlin, Germany

E-mail: h.boerner@hu-berlin.de

[b] N. Kirchner, M. Biesalski

Ernst-Berl-Institut Macromolecular and Paper Chemistry, Technical University Darmstadt, Peter-Gruenberg-Str. 8, 64287 Darmstadt, Germany

E-mail: markus.biesalski@tu-darmstadt.de

Content

1. Polymer synthesis and characterization 3
   Poly(1,4-BDT/BQA) (P1) 3
   Poly(1,3-BDT/BQA) (P2) 5
   Poly(1,2-BDT/BQA) (P3) 7
2. Additional experiments for the paper coating process 10
3. Mechanical characterization of coated paper samples 12
   Qualitative analysis 12
   Quantitative analysis 16
4. Non-mechanical characterization of coated paper samples 20
5. Filtration experiments 23
6. Recycling experiments 24

1. Polymer synthesis and characterization

Poly(1,4-BDT/BQA) (P1)

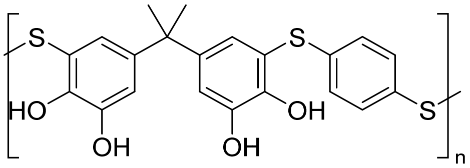

Yield: 78%

T_g = 153 °C ($T_{5\%}$ = 269 °C, T_p = 320–340 °C)

^{1}H NMR (500 MHz, DMSO-d_6):

δ = 9.55–9.10 (m, br.; 2H, OH), 8.76–8.32 (m, br.; 2H, OH), 7.08–6.32 (m, br.; 8H; BQ and DT ArH), 1.74–1.19 (m, br.; 6H; BQ aliphatic H).

FTIR:

ν = 3394 (s), 3024 (m), 2970 (s), 2869 (w), 1737 (w), 1633 (w), 1575 (m), 1506 (s), 1473 (s), 1407 (m), 1365 (s), 1278 (m), 1218 (m) 1159 (s), 1093 (w), 958 (w), 864 (w).

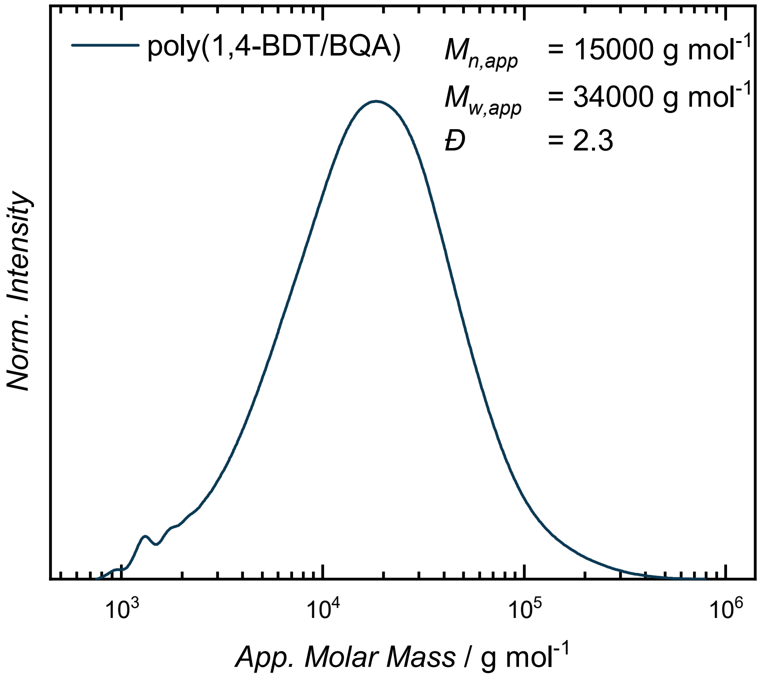

Figure S1. SEC chromatogram of **P1** prepared under standard conditions at 280 nm in THF.

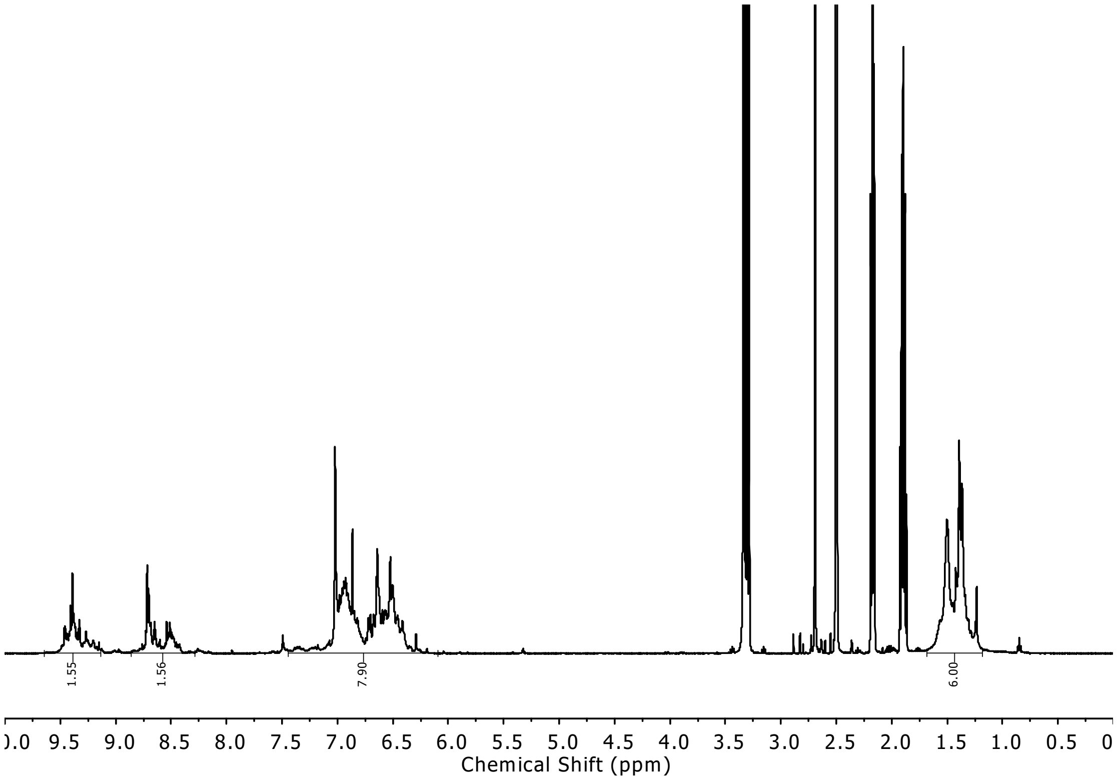

Figure S2. ^{1}H NMR spectrum of **P1** in DMSO-d_6.

Poly(1,3-BDT/BQA) (P2)

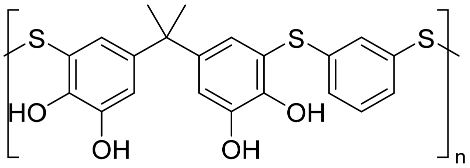

Yield: 75%

T_g = 142 °C ($T_{5\%}$ = 300 °C, T_p = 330-340 °C)

^{1}H NMR (500 MHz, DMSO-d_6):

δ = 9.59–9.09 (m, br.; 2H, OH), 8.85–8.43 (m, br.; 2H, OH), 7.20–6.37 (m, br.; 8H; BQ and DT ArH), 1.67–1.29 (m, br.; 6H; BQ aliphatic H).

FTIR:

ν = 3394 (s), 2970 (s), 2869 (w), 1737 (w), 1633 (w), 1575 (m), 1506 (s), 1473 (s), 1407 (m), 1365 (s), 1278 (m), 1218 (m) 1159 (s), 1093 (w), 958 (w), 864 (w).

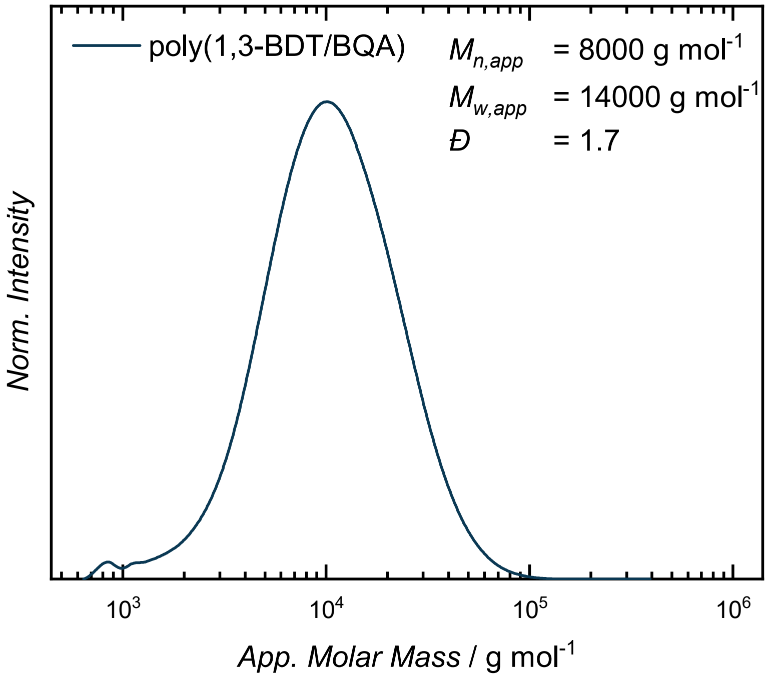

Figure S3. SEC chromatogram of **P2** prepared under standard conditions at 280 nm in THF.

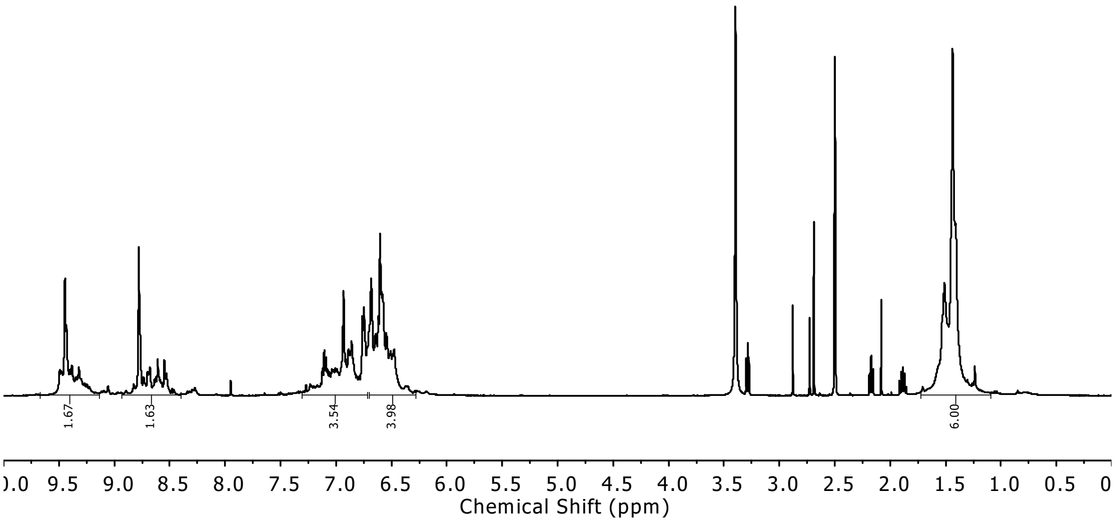

Figure S4. ^{1}H NMR spectrum of **P2** in DMSO-d_6.

Poly(1,2-BDT/BQA) (P3)

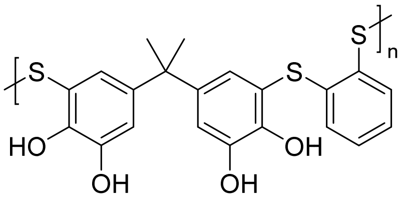

Yield: 32%

^{1}H NMR (500 MHz, DMSO-d_6):

δ = 9.58–9.19 (m, br.; 1H, OH), 8.86–8.33 (m, br.; 1H, OH), 7.07–6.64 (m, br.; 8H; BQ and DT ArH), 1.68–1.23 (m, br.; 6H; BQ aliphatic H).

FTIR:

ν = 3394 (s), 2970 (s), 2869 (w), 1737 (w), 1633 (w), 1575 (m), 1506 (s), 1473 (s), 1407 (m), 1365 (s), 1278 (m), 1218 (m) 1159 (s), 1093 (w), 958 (w), 864 (w).

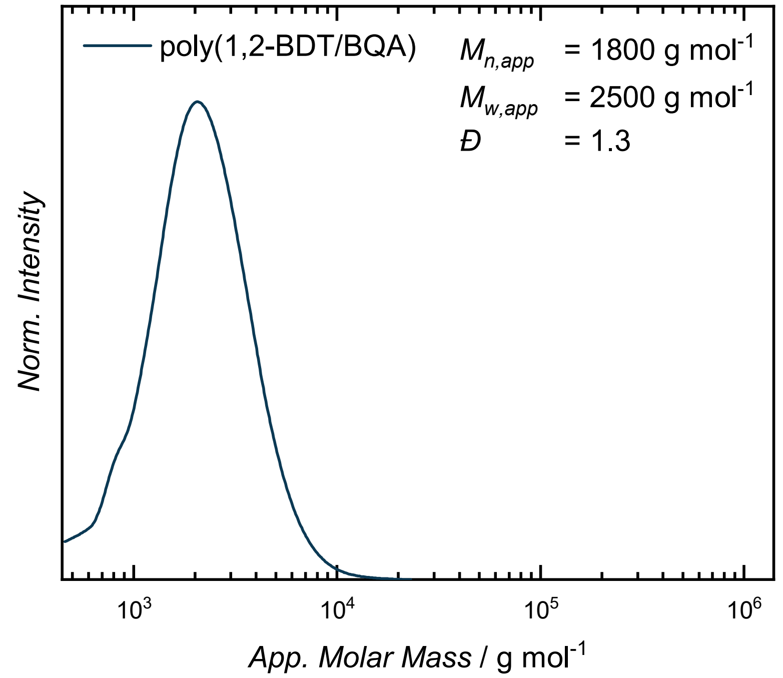

Figure S5. SEC chromatogram of **P3** prepared under standard conditions at 280 nm in THF.

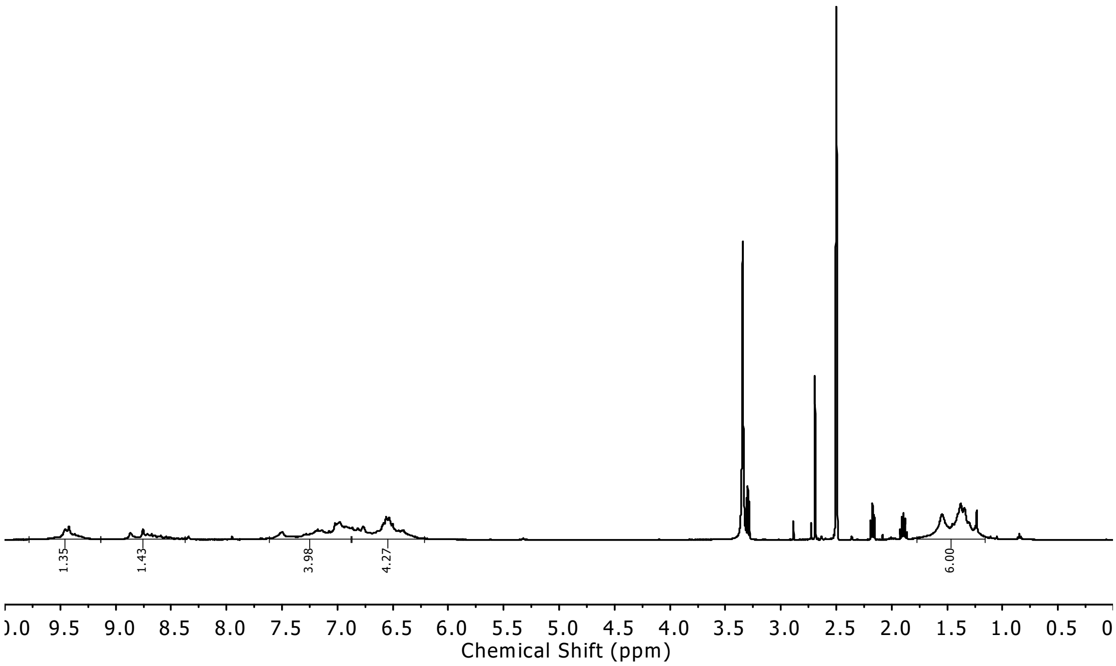

Figure S6. ^{1}H NMR spectrum of **P3** in DMSO-d6.

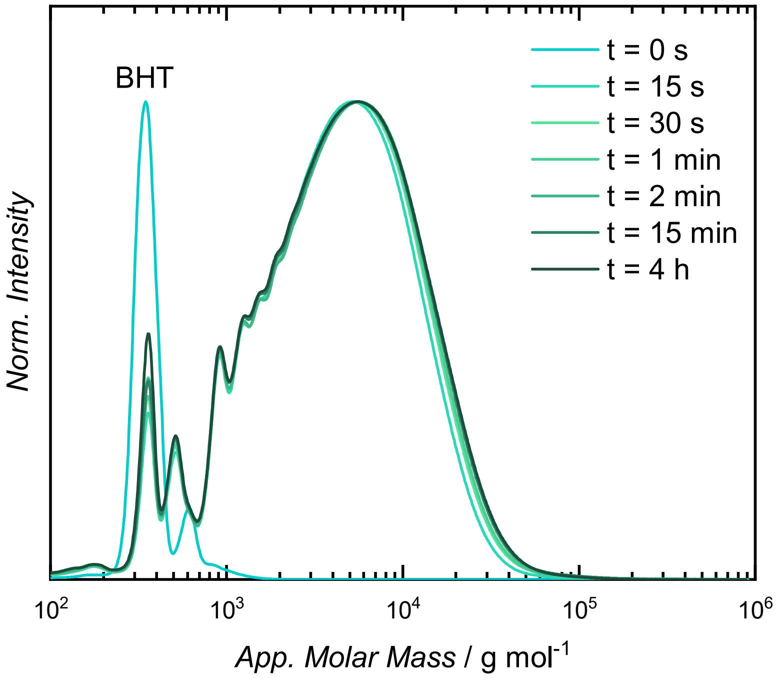

Figure S7. SEC kinetic analysis of **P1**. Samples were taken from the reaction mixture at defined time points and directly analyzed with SEC. BHT was added as internal standard.

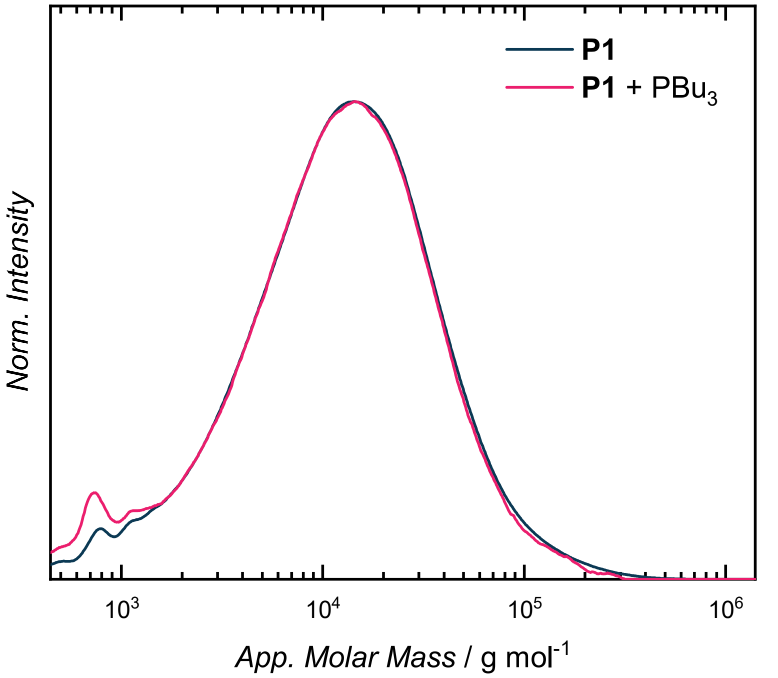

Figure S8. SEC-analysis of **P1** before (dark blue) and after treatment with an excess of tributyl phosphine overnight (red). The absence of a shift towards lower molecular weights after treatment with PBu_3 suggests negligible contribution of disulfide bonds to the polymer structure.

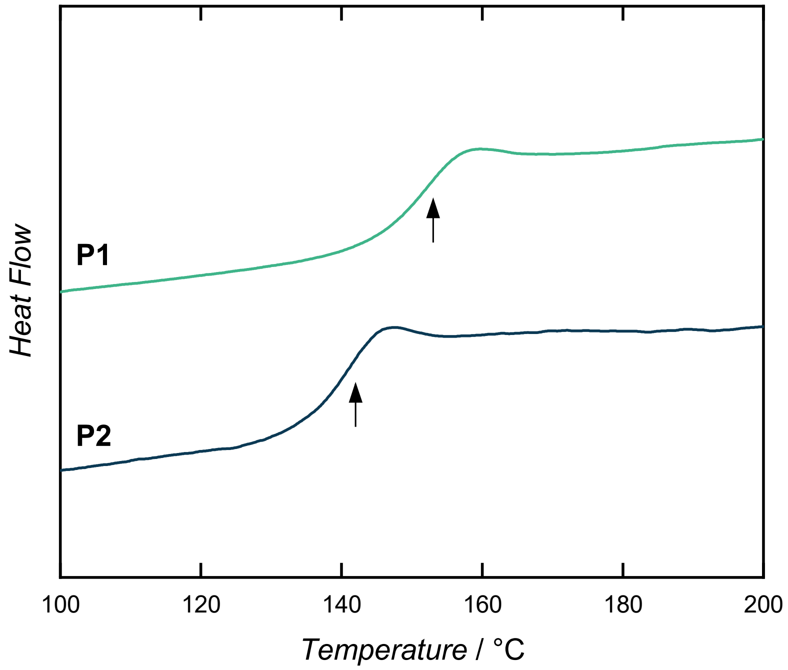

Figure S9. DSC-Analyses of poly(1,4-BDT/BQA) (**P1**, T_g = 153 °C) and poly(1,3-BDT/BQA) (**P2**, T_g = 142 °C). Poly(1,2-BDT/BQA) (**P3**) was omitted from analysis due to its low molecular weight.

2. Additional experiments for the paper coating process

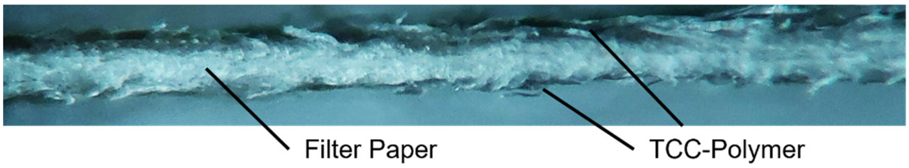

Figure S10. Cross section of filter paper after 10 soaking cycles with preformed **P1** ($M_{w,app}$ = 34000 g mol^{-1}, $Đ$ = 2.3) in DMF (polymer content: 32 wt.-%). The greyish-brown **P1** can be seen accumulating at the easily accessible top and bottom surfaces of the paper, leaving the inside of the paper largely uncovered and white.

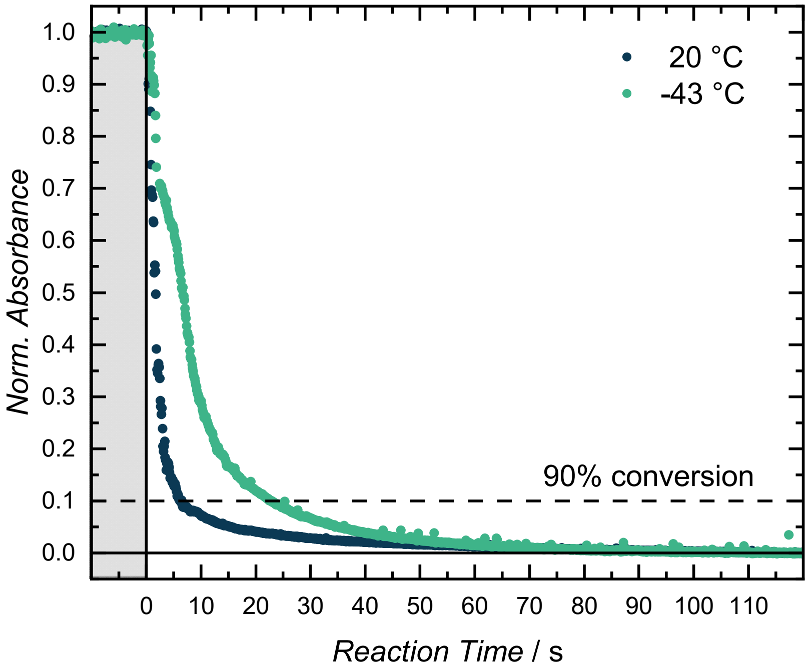

Figure S11. Discoloration kinetics for the production of polymer **P1**. A conversion of 90% is reached after 6 s at 20 °C and after 23 s at -43 °C. Complete discoloration is observed at around 70 s at -43 °C.

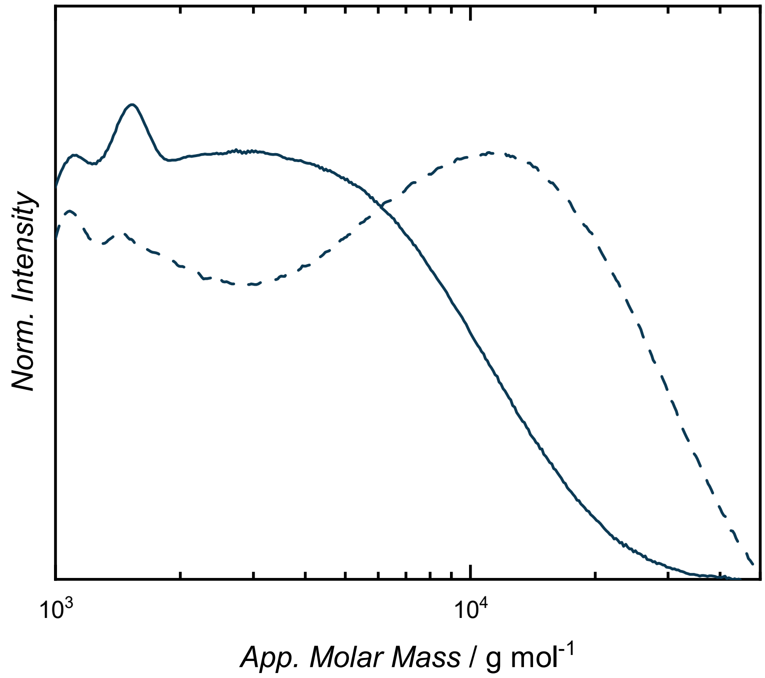

Figure S12. SEC-analysis of **P1**, extracted from **P1@FP[5]** using DMF (solid line), as well as **P1**, obtained from the supernatant dip-coating solution (dashed line). The discrepancy at high molecular weights suggests limitations in generating long polymer chains within the paper sample.

3. Mechanical characterization of coated paper samples

Qualitative analysis

Table S1. Overview over the samples analyzed in the qualitative bending measurements. 100% represents the stiffness of pristine filter paper.

Coating (Cycles)	Coating Content [wt.-%]	Mp [g mol^{-1}][a]	Stiffness [%]
poly(1,2-BDT/BQA) (5)	31.8	700	227 ± 12
poly(1,3-BDT/BQA) (5)	31.5	2000	256 ± 6
poly(1,4-BDT/BQA) (5)	29.0	3000	258 ± 15
poly(1,4-BDT/BQA) (10)	47.6	n.d.	431 ± 10
poly(1,4-BMT/BQA) (5)	29.8	2000	106 ± 9
poly(EDET/BQA) (5)	23.8	2000	10.7 ± 14.4
BPA (5)	33.7	N/A	-6.9 ± 27.4

a: Peak molar mass of the dominating mass distribution in the DMF-extract, determined by SEC.

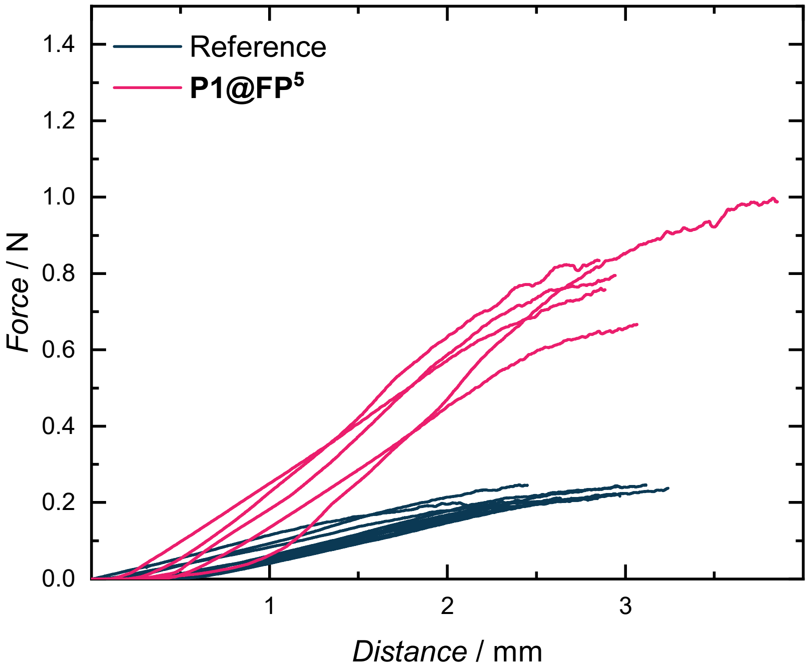

Figure S13. Qualitative mechanical analysis of **P1@FP^5** (red) and a reference sample set of unmodified MN 616 (dark blue).

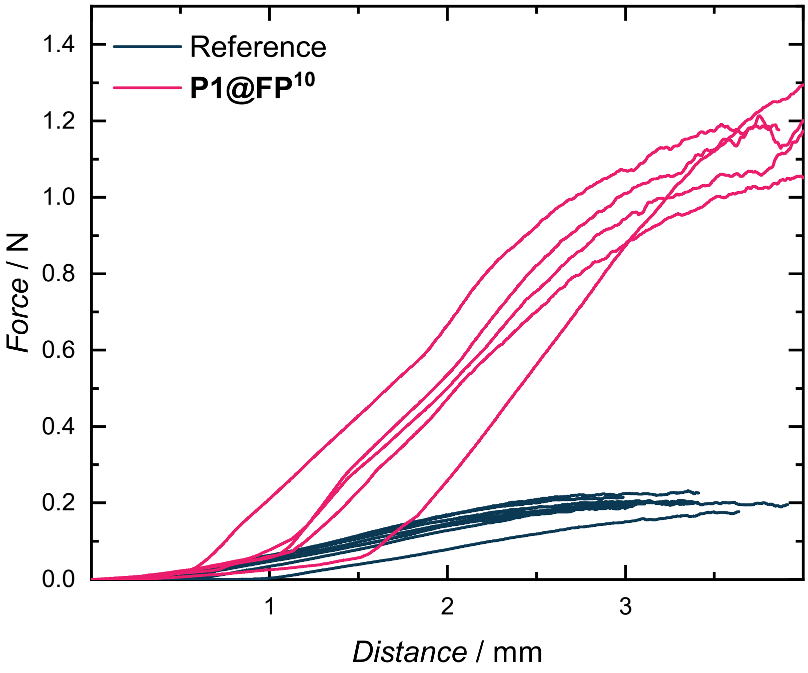

Figure S14. Qualitative mechanical analysis of **P1@FP^{10}** (red) and a reference sample set of unmodified MN 616 (dark blue).

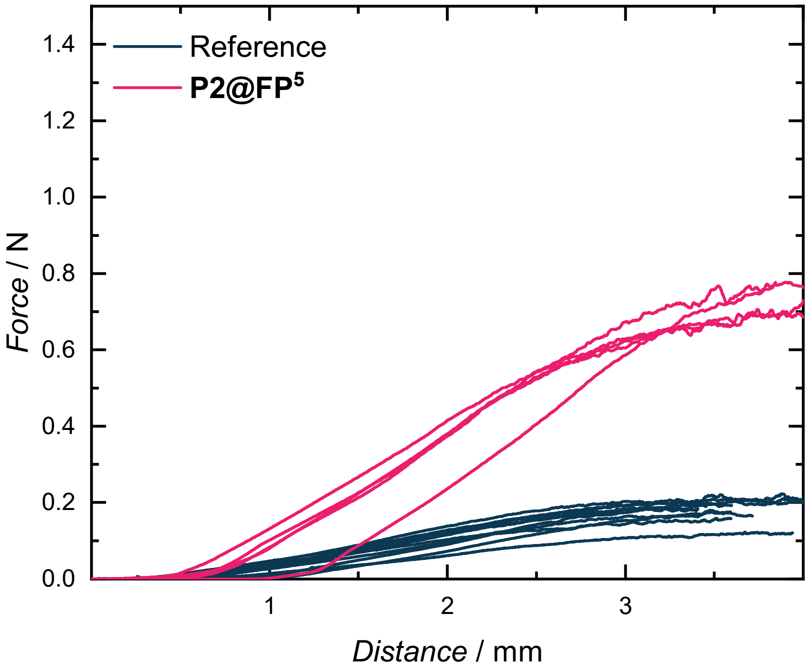

Figure S15. Qualitative mechanical analysis of **P2@FP**[5] (red) and a reference sample set of unmodified MN 616 (dark blue).

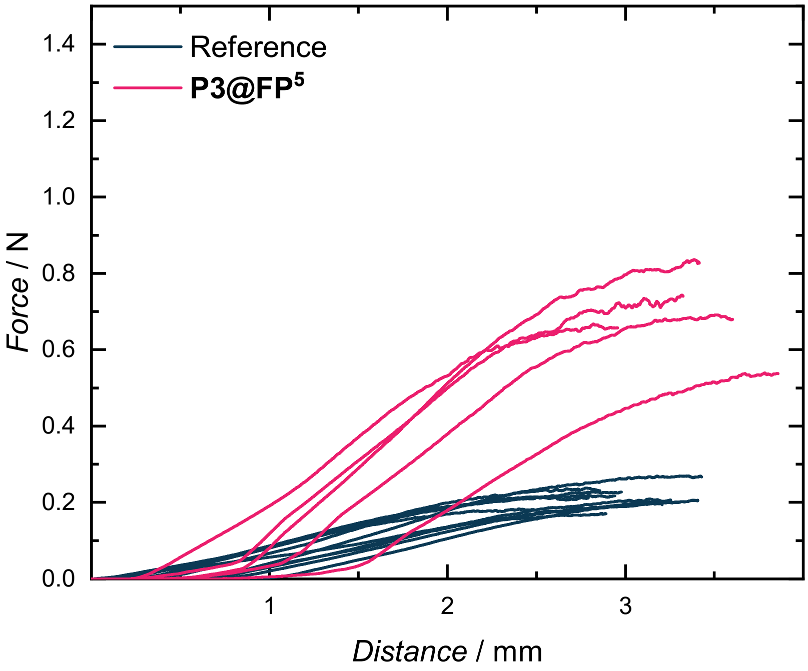

Figure S16. Qualitative mechanical analysis of **P3@FP**[5] (red) and a reference sample set of unmodified MN 616 (dark blue).

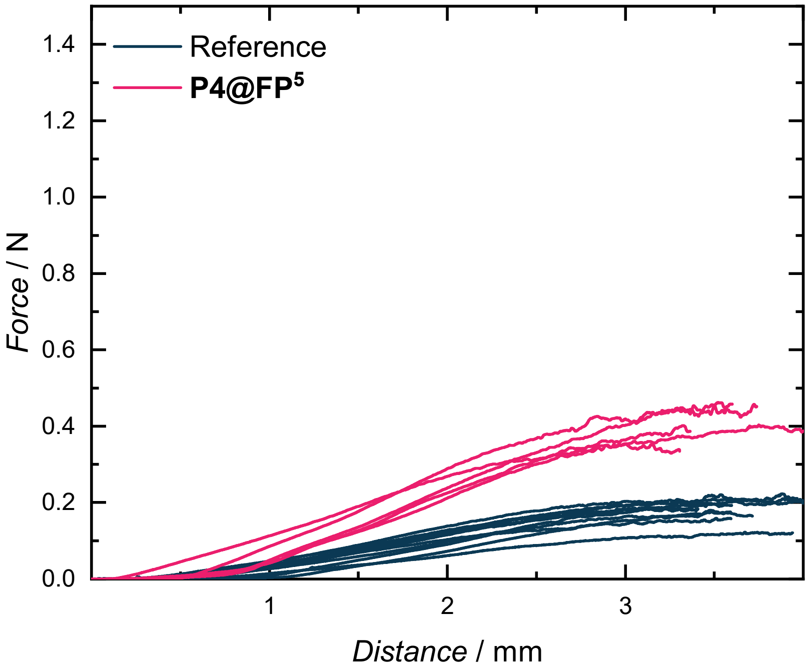

Figure S17. Qualitative mechanical analysis of **P4@FP⁵** (red) and a reference sample set of unmodified MN 616 (dark blue).

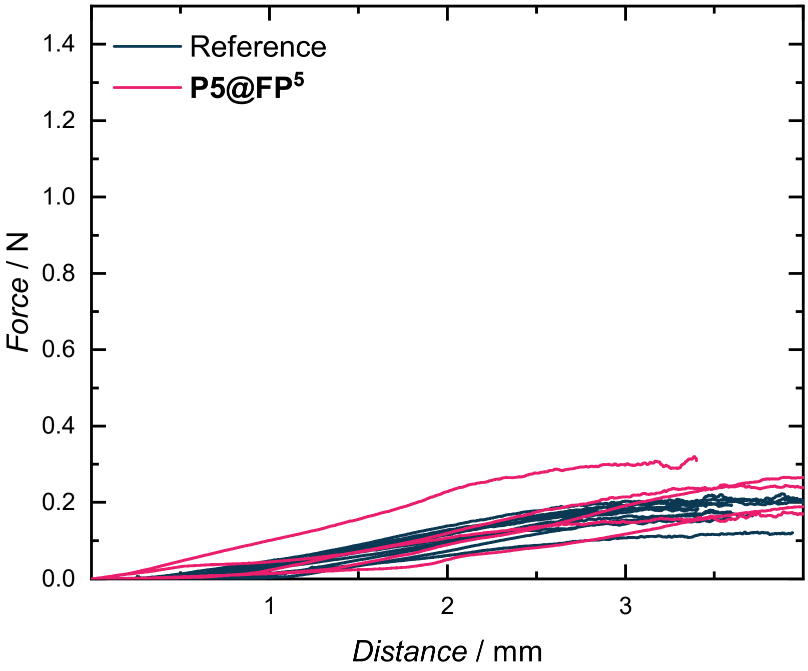

Figure S18. Qualitative mechanical analysis of **P5@FP⁵** (red) and a reference sample set of unmodified MN 616 (dark blue).

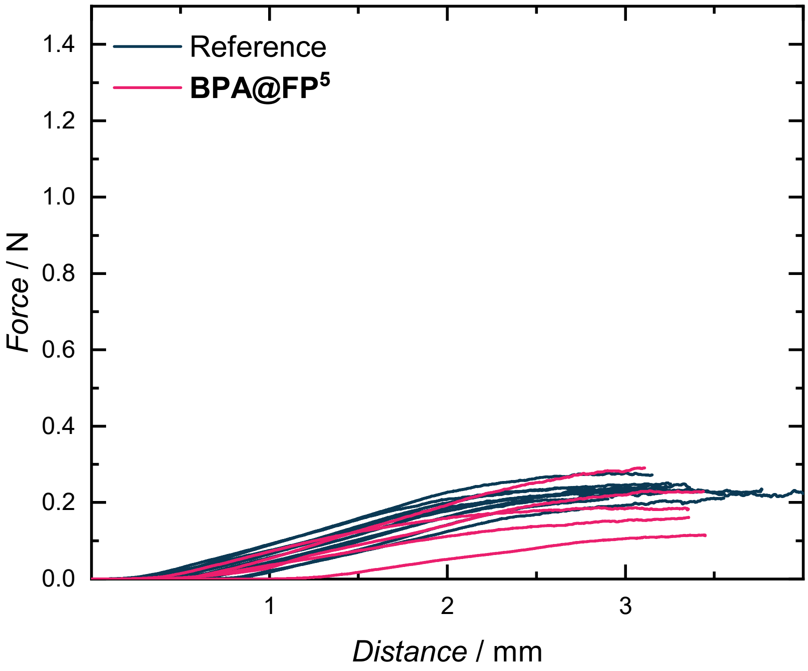

Figure S19. Qualitative mechanical analysis of **BPA@FP**[5] (red) and a reference sample set of unmodified MN 616 (dark blue).

Quantitative analysis

Table S2. Overview of the samples analyzed in quantitative tensile tests and contact angle measurements. Polymer content and grammage were calculated based on the total weight of all 10 paper strips used for tensile measurements and the volume of a single 18 cm^2 strip. Dry and wet tensile indices were calculated by normalizing the tensile strength to the grammage.

Sample	Polymer Content [wt.-%]	m (set) [g]	Grammage [g m^{-2}]	Dry Tensile Index [Nm g^{-1}]	Wet Tensile Index [Nm g^{-1}]	Relative Wet Strength [%]	Water Contact Angle [°]
**FP**	1.5209	0	85[a]	0.47 ± 0.02	0.06 ± 0.00	12.8 ± 0.5	0[b]
**P1@FP**[1]	1.6650	8.7	92.5	0.49 ± 0.03	0.11 ± 0.01	22.5 ± 2.5	48.5 ± 11.8
**P1@FP**[4]	2.0378	23.4	113.2	0.65 ± 0.07	0.25 ± 0.02	38.5 ± 5.2	105.9 ± 4.8
**P1@FP**[7]	2.4186	35.3	134.4	0.75 ± 0.09	0.36 ± 0.09	48.0 ± 13.3	122.1 ± 1.7
**P1@FP**[10]	2.7252	42.4	151.4	0.82 ± 0.05	0.39 ± 0.03	47.6 ± 4.7	126.8 ± 4.1

a: as specified by the manufacturer; b: arbitrarily set to 0°, as water immediately gets fully imbibed.

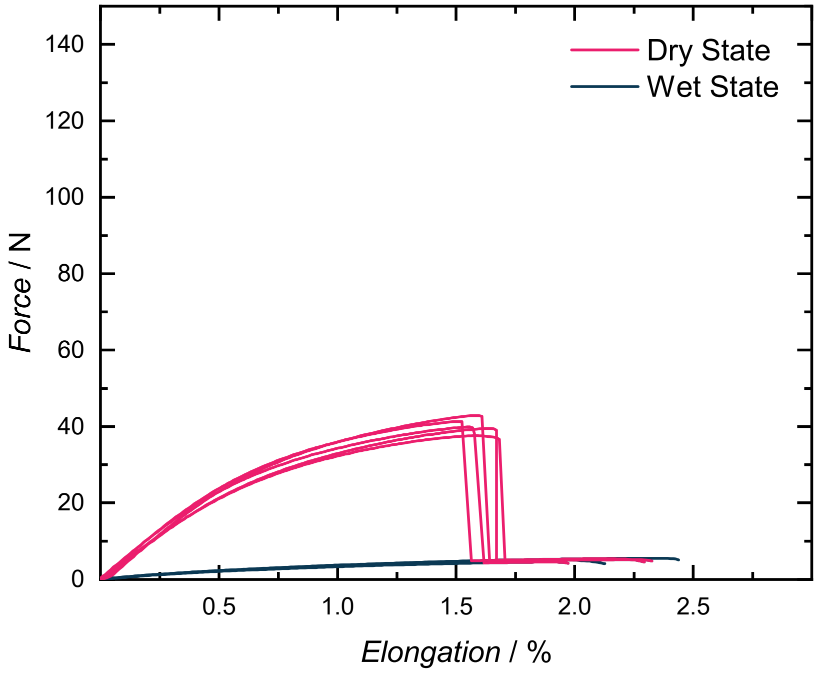

.

Figure S20. Quantitative mechanical analysis of the **FP** reference in the dry (red) and the wet state (dark blue).

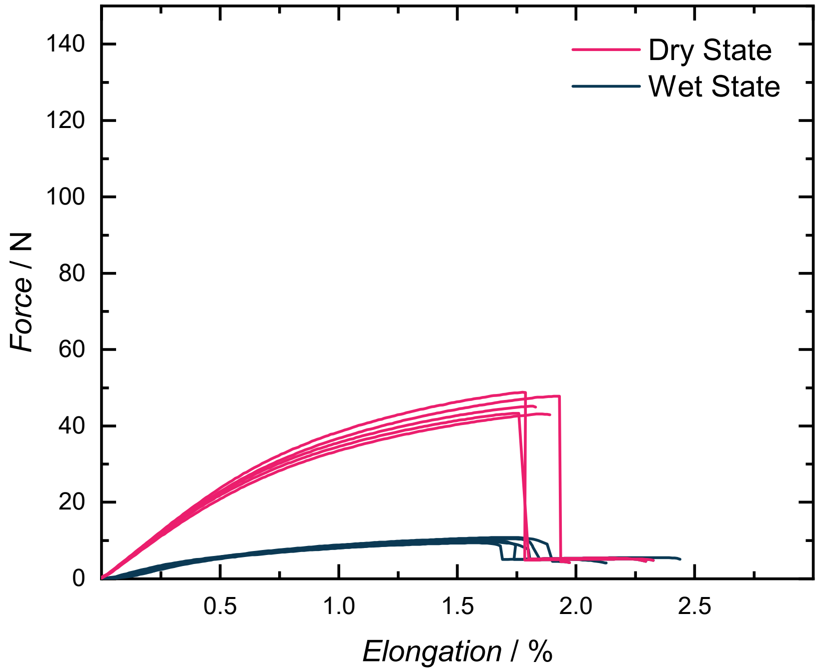

Figure S21. Quantitative mechanical analysis of **P1@FP[1]** in the dry (red) and the wet state (dark blue).

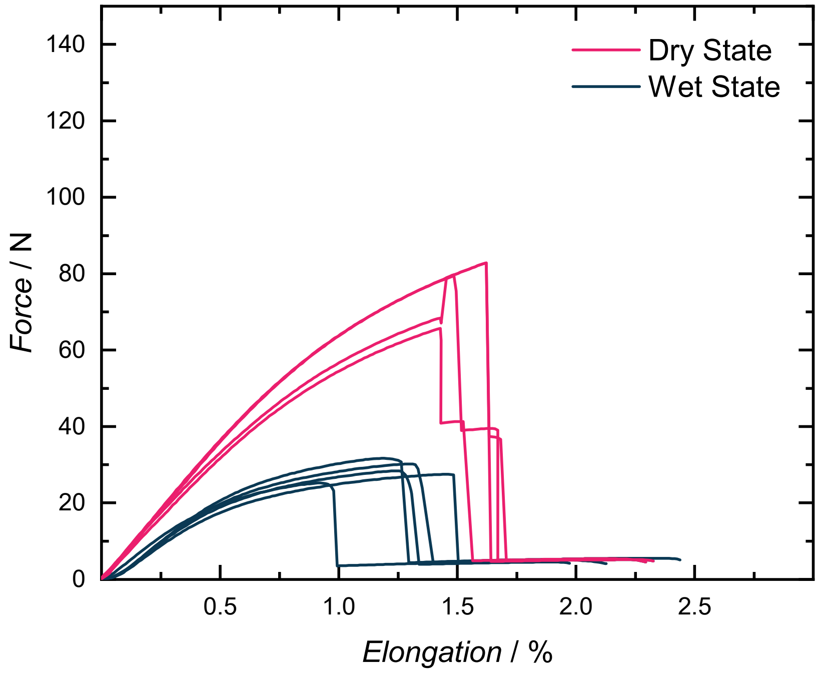

Figure S22. Quantitative mechanical analysis of **P1@FP[4]** in the dry (red) and the wet state (dark blue). Only four measurements could be taken in dry state as one of the papers was already damaged before the tensile measurement.

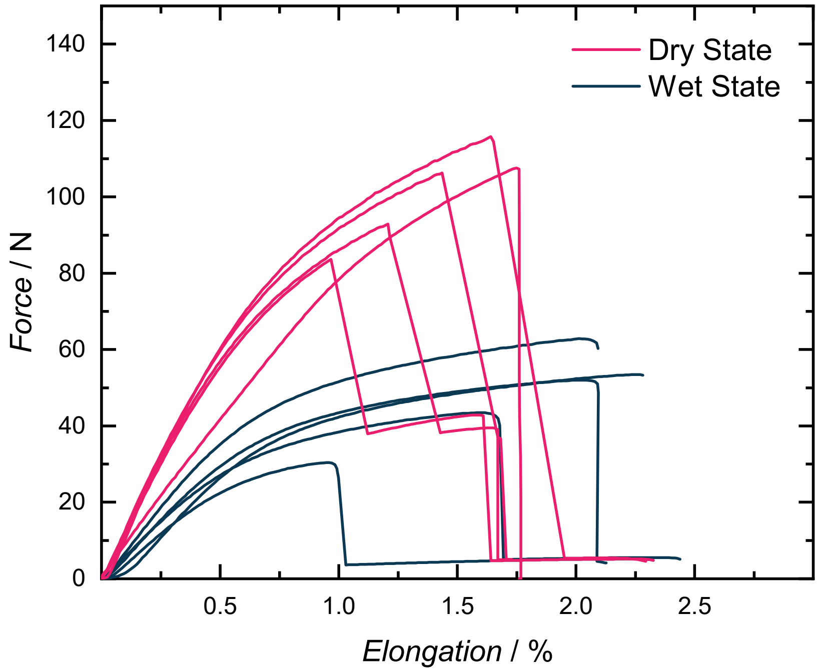

Figure S23. Quantitative mechanical analysis of **P1@FP[7]** in the dry (red) and the wet state (dark blue).

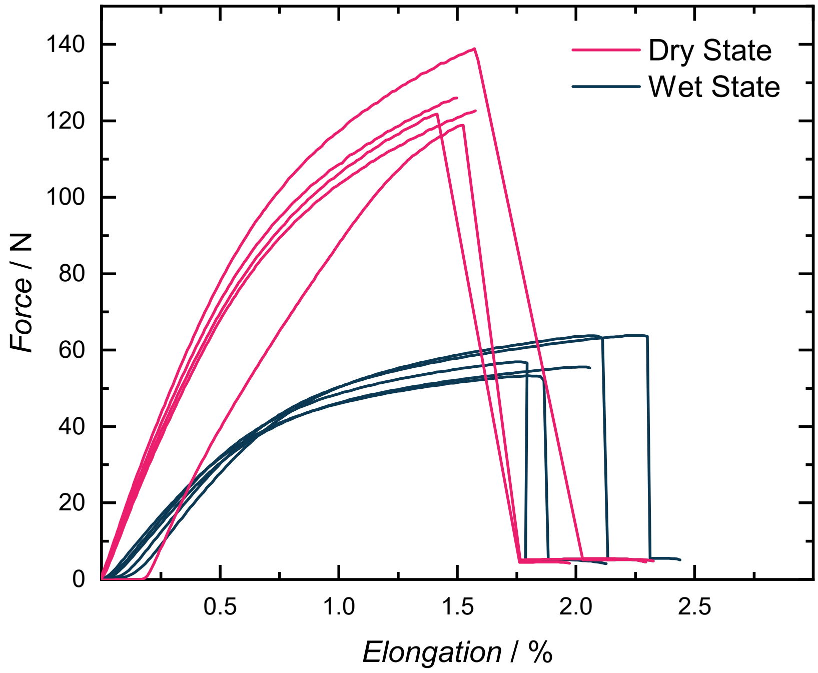

Figure S24. Quantitative mechanical analysis of **P1@FP^{10}** in the dry (red) and the wet state (dark blue).

4. Non-mechanical characterization of coated paper samples

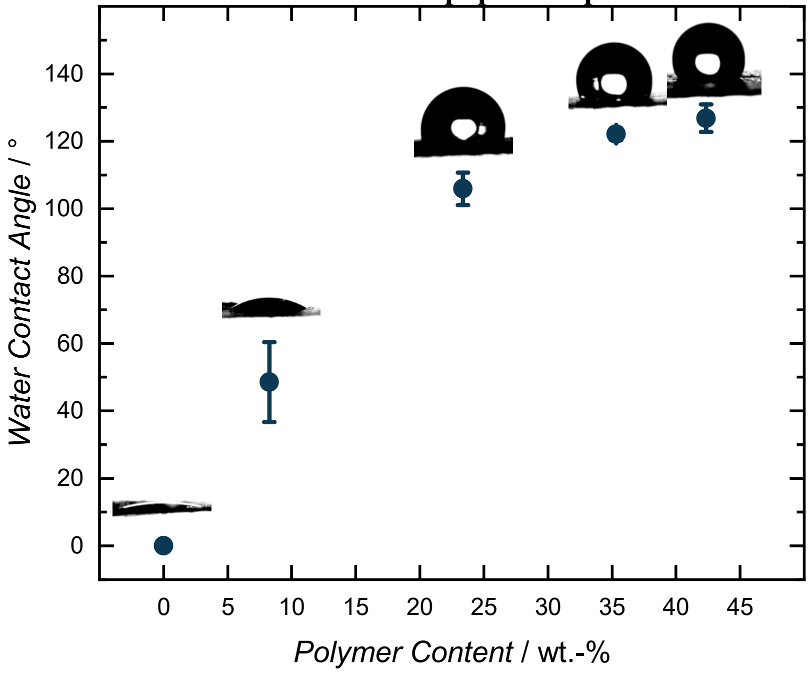

Figure S25. Contact angle measurements of **FP**, **P1@FP[1]**, **P1@FP[4]**, **P1@FP[7]** and **P1@FP[10]**. Since water is immediately absorbed by **FP**, the contact angle has been arbitrarily set to 0°.

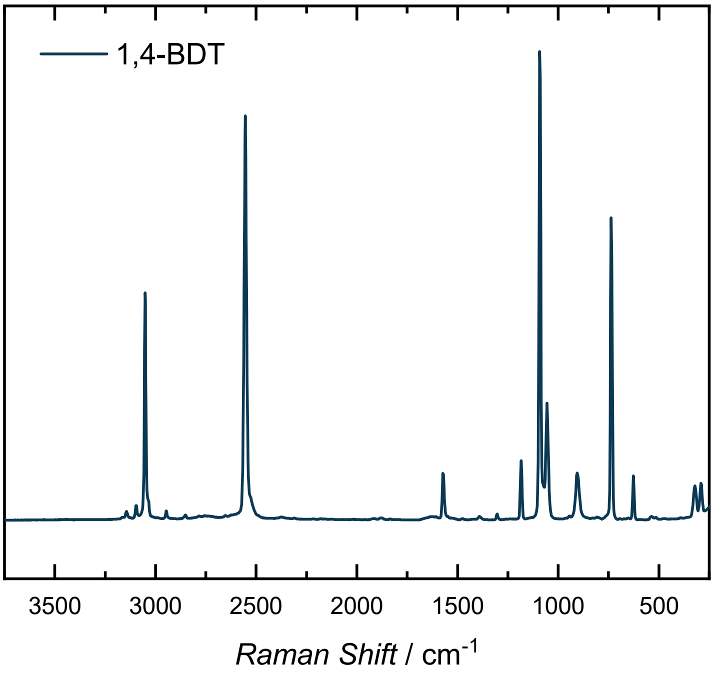

Figure S26. Raman-spectrum of solid 1,4-BDT.

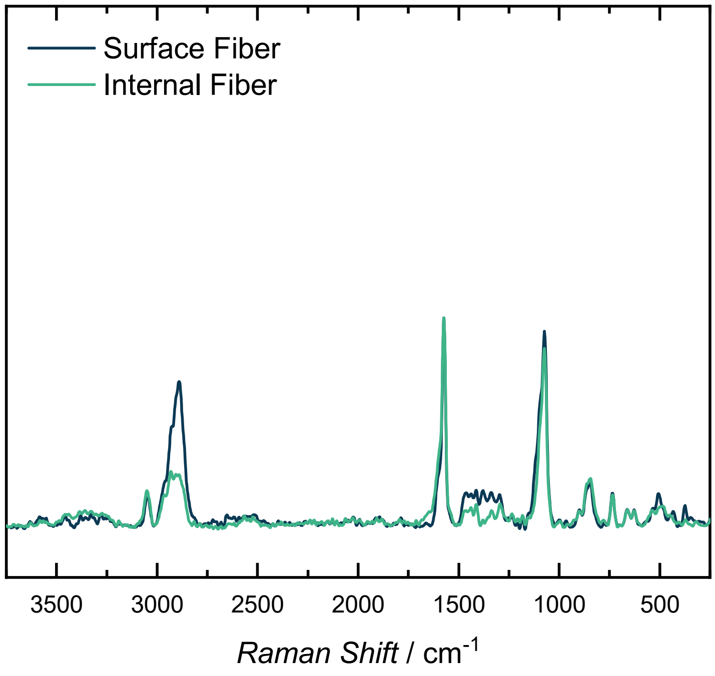

Figure S27. Raman-spectra of **P1@FP**[10] taken from a surface fiber (dark blue) and a randomly selected fiber from the paper bulk (green). The close match between the two spectra indicates the homogeneous distribution of **P1** throughout the paper sheet.

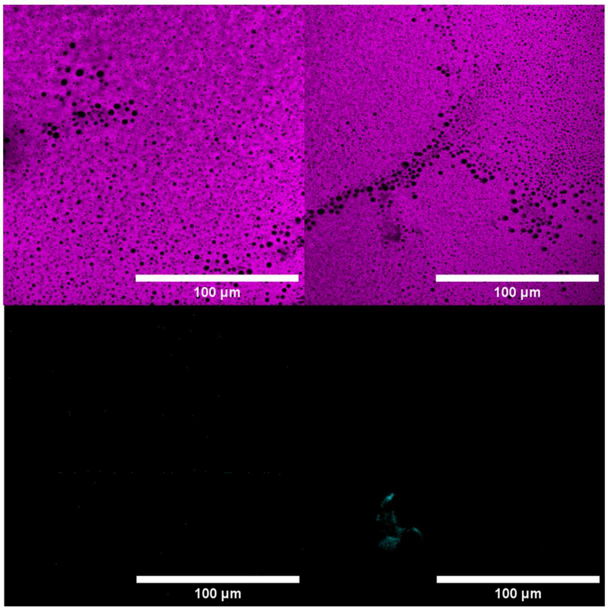

Figure S28. CLSM micrographs of a drop-cast **P1***-film on a glass substrate, before (left) and after (right) staining with Calcofluor White. The TCC-polymer fluorescence is shown in magenta (top) and the Calcofluor White fluorescence is shown in cyan (bottom). No staining of **P1*** with Calcofluor White is observed.

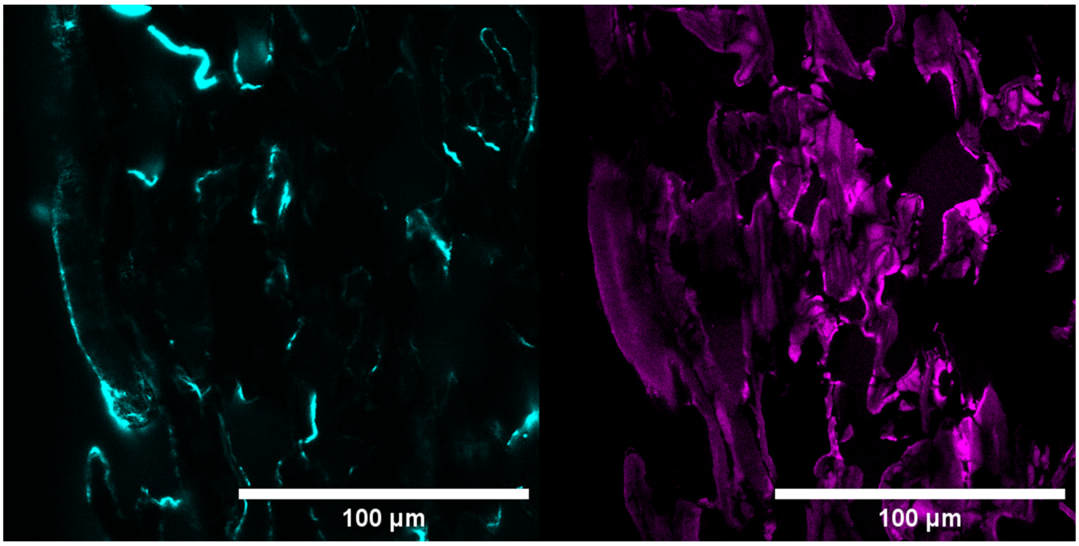

Figure S29. CLSM-micrographs of a cross section of **P1*@FP**^{10} with free cellulose appearing in cyan (left) and TCC-polymer appearing in pink (right). While Calcofluor White staining only reaches fiber surfaces, producing outlines of the fibers, the polymer appears to be present within the fibers, producing colored areas. Moreover, no polymer is observed outside the fibers, indicating that no obstruction of the pore system has taken place. Due to the high polymer coverage, Calcofluor White cellulose staining does not cover all fiber surfaces.

5. Filtration experiments

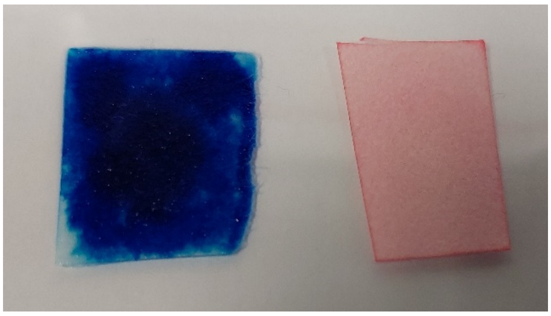

Figure S30. Pristine filter paper after wetting with water (left) and cyclohexane (right). Both liquids are readily absorbed. Water has been dyed blue with methylene blue and cyclohexane has been dyed red with Sudan IV.

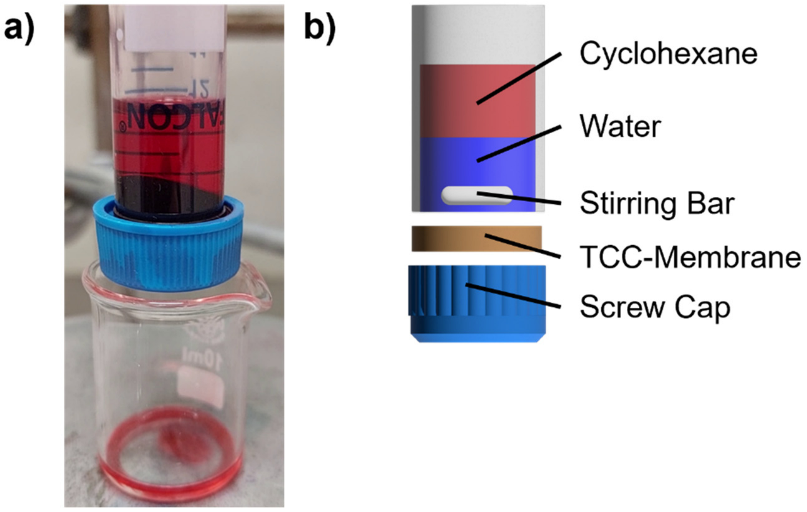

Figure S31. Vertical solvent/water-separation experiments. Cyclohexane gets selectively percolated, bypassing the aqueous phase (a). Schematic of the filtration setup (b).

6. Recycling experiments

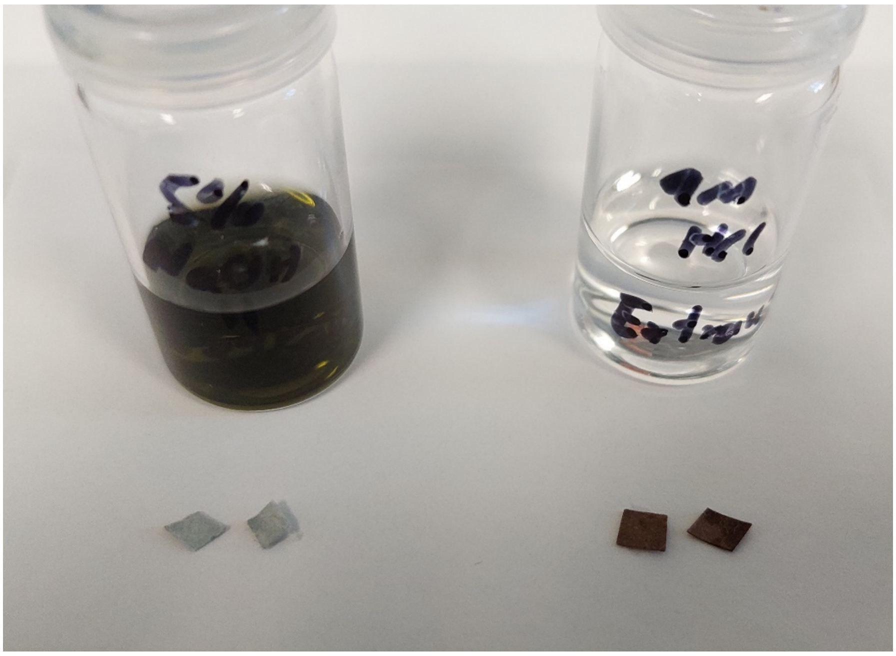

Figure S32. P1@FP^{10} after washing (2 h + 1 h) with 1.25 M NaOH (left) and 1 M HCl (right). While the basic solution turned dark green and a washout of polymer could be observed, the acid wash did not visibly affect the paper sample.

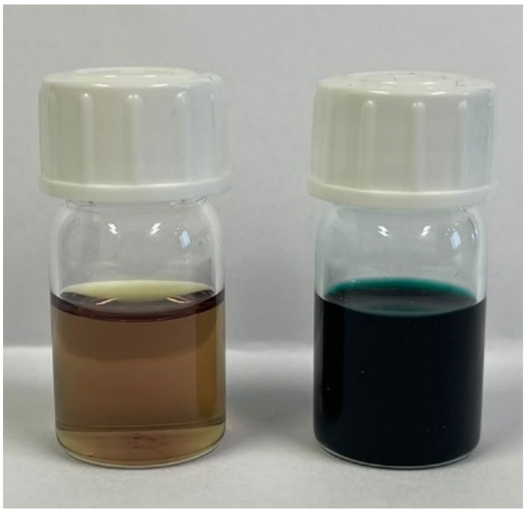

Figure S33. Solution of BQA in DMF before (left) and after (right) the addition of one drop of 1 M NaOH.

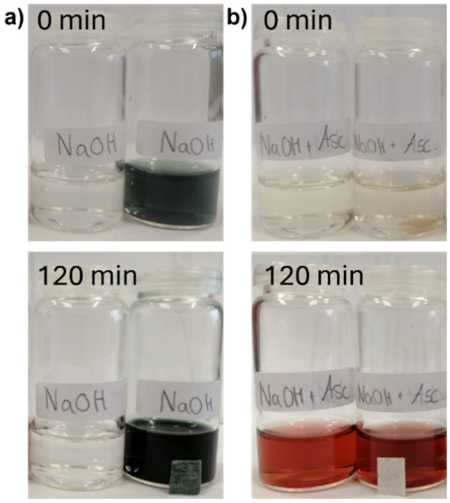

Figure S34. Solutions of 1.25 M NaOH (a) and 0.22 M ascorbic acid/1.25 M NaOH (b) used in the extraction study. The pictures show the solutions without (left) and with a piece of **P1@FP^{10}** added (right) immediately at the start of the experiment as well as after 2 h with the extracted paper samples placed in front of the vials. The discoloration of both the pure and the extractive ascorbate solution suggests that the red color stems from alkaline degradation of ascorbic acid rather than from the extracted TCC-polymers.[1]

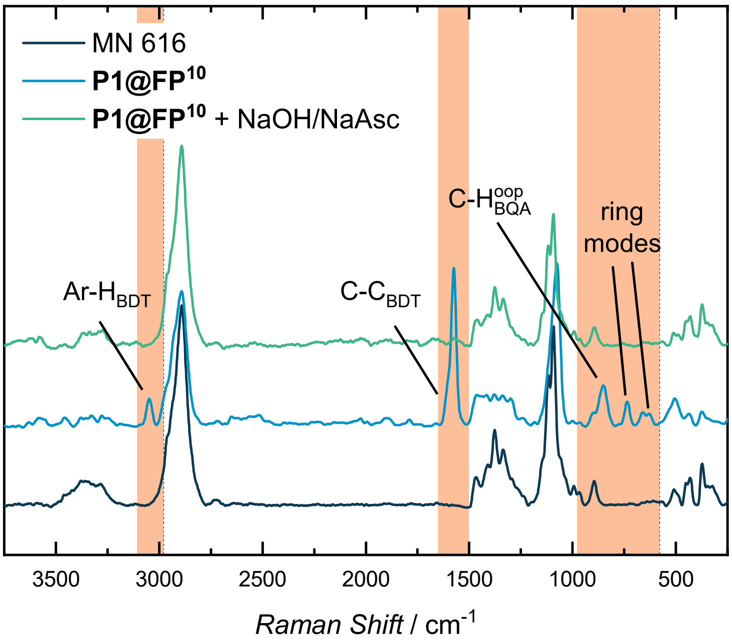

Figure S35. Raman-spectra of pristine filter paper (dark blue) as well as **P1@FP^{10}** before (light blue) and after extraction with NaOH/NaAsc (green). Peaks corresponding to **P1** are highlighted in orange.

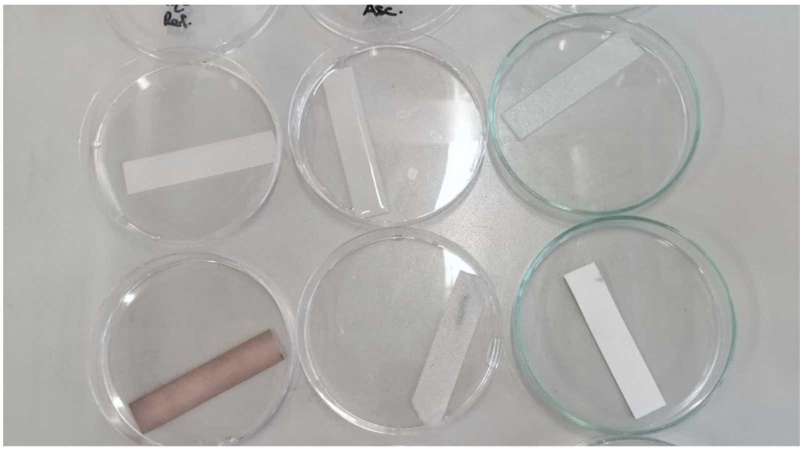

Figure S36. Strips of pristine filter paper (top) and **P1@FP[10]** (bottom) used in the recycling experiments after their washing with deionized water (left), 1.25 M NaOH and 0.22 M ascorbic acid (middle) and DMF (right).

Table S3. Overview over the gravimetric analysis of the **P1@FP[10]** samples washed with different solvents, their dry weight before and after washing and the calculated washing efficiency η_{wash}.

**Solvent**	$m_{initial}$ **[mg]**	m_{final} **[mg]**	η_{wash} **[%]**
Deionized water	115.0	110.8	8.6
Deionized water	111.1	108.9	4.7
DMF	100.8	62.0	90.8
DMF	100.0	61.0	92.0
NaOH + Asc	102.4	66.7	82.2
NaOH + Asc	102.4	62.0	93.0

Table S4. Overview over the recycling experiments and the corresponding dispersibility scores (DS).[2]

	Solvent		
Sample	Deionized water	NaOH + Asc	DMF
**FP**	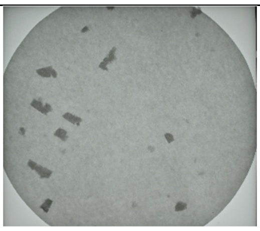	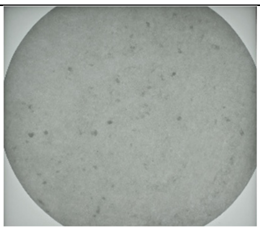	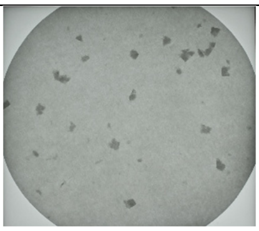
dispersibility score	5	9	6
**FP**	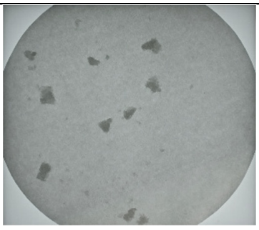	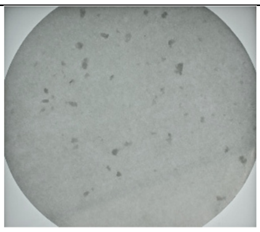	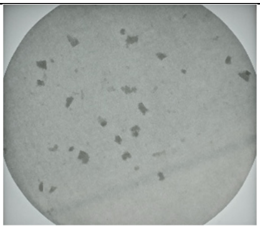
dispersibility score	5	8	7
**P1@FP^{10}**	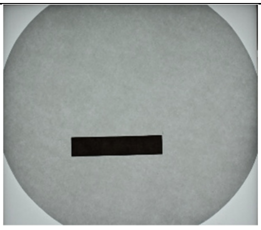	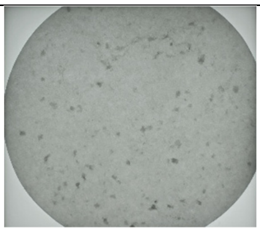	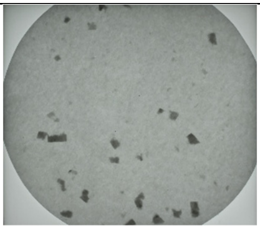
dispersibility score	0	8	6
**P1@FP^{10}**	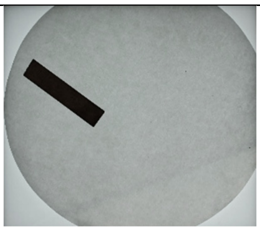	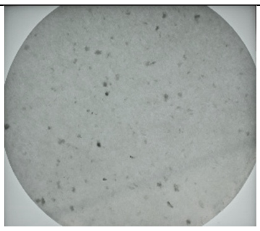	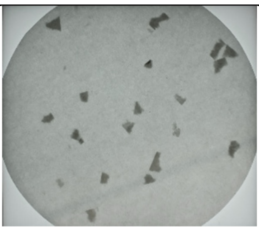
dispersibility score	0	9	5

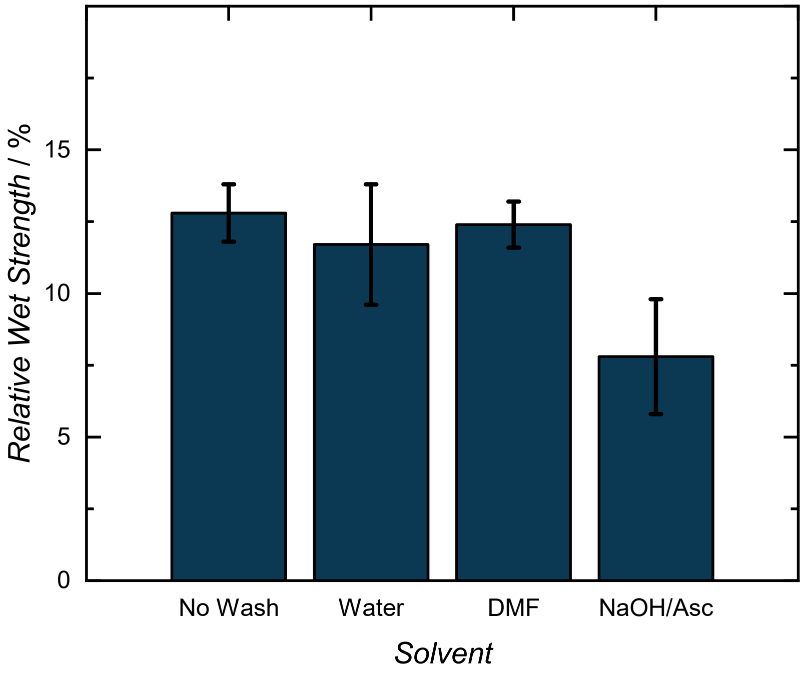

Figure S37. Relative wet strength of **FP** samples after extraction with deionized water, DMF, or 1.25 M NaOH/0.22 M ascorbic acid for 2 h. While washing with water or DMF did not affect the relativ wet strength compared to untreated **FP**, NaOH/Asc treatment resulted in a moderate decrease.

Table S5. Overview of samples analyzed in quantitative tensile tests after the washing procedure. Dry and wet tensile indices were normalized to a grammage of 85 g m^{-2}.

**Solvent**	**Dry Tensile Index** [$Nm\ g^{-1}$]	**Wet Tensile Index** [$Nm\ g^{-1}$]	**Relative Wet Strength** [%]
**No Wash**	0.47 ± 0.02	0.060 ± 0.004	12.8 ± 1.0
**Water**	0.46 ± 0.03	0.054 ± 0.009	11.7 ± 2.1
**DMF**[a]	0.29 ± 0.01	0.036 ± 0.002	12.4 ± 0.8
**NaOH/ Asc**	0.41 ± 0.02	0.032 ± 0.008	7.8 ± 2.0

a: Sample measured in cross direction (CD) instead of machine direction (MD). This resulted in lower absolute tensile indices, but the relative wet strength remains comparable.

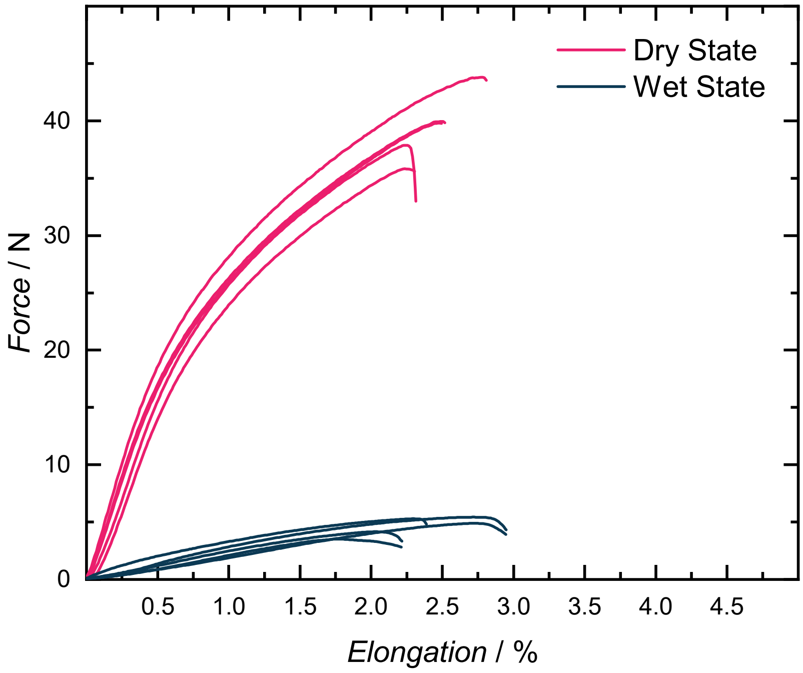

Figure S38. Quantitative tensile measurements of **FP** extracted with deionized water in dry (red) and wet (dark blue) state, measured in machine direction (MD).

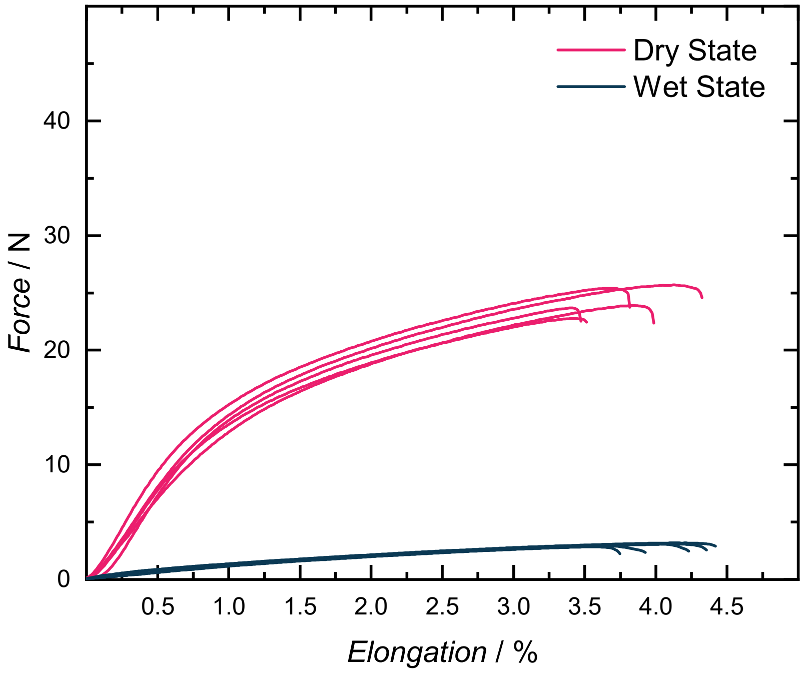

Figure S39. Quantitative tensile measurements of **FP** extracted with DMF in dry (red) and wet (dark blue) state, measured in cross direction (CD).

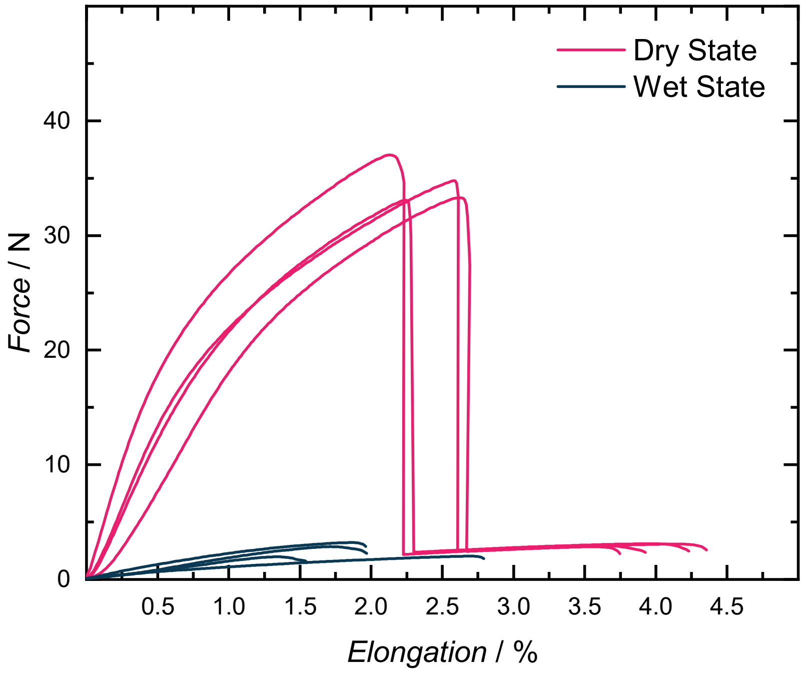

Figure S40. Quantitative tensile measurements of **FP** extracted with 1.25 M NaOH and 0.22 M ascorbic acid in dry (red) and wet (dark blue) state, measured in machine direction (MD).

REFERENCES

(1) Yuan, J.-P.; Chen, F. Degradation of Ascorbic Acid in Aqueous Solution. *J. Agric. Food. Chem.* **1998**, *46* (12), 5078–5082. DOI: 10.1021/jf9805404.

(2) Pfennich, A. C.; Schoeffmann, E. A.; Lammer, H.; Hirn, U. Water-dispersible paper for packaging applications – balancing material strength and dispersibility. *Nord. Pulp Pap. Res. J.* **2023**, *38* (4), 521-532. DOI: 10.1515/npprj-2023-0048.
